# Relationships between sodium, fats and carbohydrates on blood pressure, cholesterol and HbA1c: an umbrella review of systematic reviews

**DOI:** 10.1136/bmjnph-2023-000666

**Published:** 2024-03-21

**Authors:** Penny Breeze, Katie Sworn, Ellen McGrane, Sarah Abraham, Anna Cantrell

**Affiliations:** 1 Division of Population Health, The University of Sheffield, Sheffield, UK; 2 Institute of Nursing Science Clinical-Theoretical Institute of the University Hospital, Albert-Ludwigs-Universität Freiburg, Freiburg im Breisgau, Baden-Württemberg, Germany; 3 University of Sheffield, Sheffield, UK

**Keywords:** Blood pressure lowering, Diabetes mellitus, Lipid lowering, Metabolic syndrome

## Abstract

**Background:**

The relationship between nutrition and health is complex and the evidence to describe it broad and diffuse. This review brings together evidence for the effect of nutrients on cardiometabolic risk factors.

**Methods:**

An umbrella review identified systematic reviews of randomised controlled trials and meta-analyses estimating the effects of fats, carbohydrates and sodium on blood pressure, cholesterol and haemoglobin A1c (HbA1c). Medline, Embase, Cochrane Library and Science Citation Index were search through 26 May 2020, with supplementary searches of grey literature and websites. English language systematic reviews and meta-analyses were included that assessed the effect of sodium, carbohydrates or fat on blood pressure, cholesterol and HbA1c. Reviews were purposively selected using a sampling framework matrix. The quality of evidence was assessed with A MeaSurement Tool to Assess systematic Reviews 2 (AMSTAR2) checklist, evidence synthesised in a narrative review and causal pathways diagram.

**Results:**

Forty-three systematic reviews were included. Blood pressure was significantly associated with sodium, fibre and fat. Sodium, fats and carbohydrates were significantly associated with cholesterol. Monounsaturated fat, fibre and sugars were associated with HbA1c.

**Conclusion:**

Multiple relationships between nutrients and cardiometabolic risk factors were identified and summarised in an accessible way for public health researchers. The review identifies associations, inconsistencies and gaps in evidence linking nutrition to cardiometabolic health.

**WHAT IS ALREADY KNOWN ON THIS TOPIC**

- There is extensive research describing the associations between diet and cardiometabolic risk factors. However, the evidence from high-quality systematic reviews to describe these effects is diverse, overlapping and dispersed making it challenging for researchers to access up-to-date evidence across all relevant nutritional markers and cardiometabolic outcomes.

**WHAT THIS STUDY ADDS**

- This review brings together evidence across nutrients to provide consistent quantitative estimates of the associations between nutritional intake and cardiometabolic risk.

**HOW THIS STUDY MIGHT AFFECT RESEARCH, PRACTICE OR POLICY**

- This review supports the evaluation of public health policies targeting behavioural aspects of diet, particularly for population-level interventions, where randomised controlled trial evidence cannot easily be collected. The review provides a single resource that brings together evidence across nutrients and cardiometabolic risks to develop the capacity to evaluate public health dietary policies.

## Introduction

Suboptimal diets are estimated to be responsible for 11 million deaths globally, more than smoking tobacco.^1^ Diet is a major contributory factor in the incidence of diabetes, cardiovascular disease and other non-communicable diseases, which cause a major burden on healthcare resources. Cardiovascular disease alone is estimated to be €210 bn/year in Europe, of which the majority (€111 bn) is healthcare costs, and the remainder is productivity losses (€45 bn) and informal care (€45 bn).^2^

In order to evaluate the effectiveness of dietary policies, it is necessary to have a reliable evidence base to describe the health benefits of dietary changes, particularly if the changes in nutritional intake have competing health outcomes, for example, if the policy reduces sugar intake, but increased salt. Population-level dietary public health policies are often evaluated in modelling studies to estimate the potential benefits, where the health effects cannot be easily observed. Modelling studies often make simplifying assumptions such as assuming all health benefits are captured by a single risk factor between diet and health, such as salt,^3^ fruit and vegetables,^4^ or calories.^5 6^ While economic evaluations have modelled a variety of associations between nutrition to health,^7^ few have modelled multiple nutritional components and captured food substitutions. Simulating substitutions to other food items is important to capture the overall benefit of a policy and any mitigating unintended consequences.

There is a large and rich literature describing the impacts of diet on cardiometabolic health, and cardiovascular disease. Systematic reviews have synthesised evidence for differing levels of individual nutrient groups, such as sodium,^8^ or carbohydrates, on the risk of cardiovascular disease.^9^ Changes to nutritional intake in real-world contexts often take the form of diets, which consist of multiple nutrient adjustments that impact the same cardiovascular outcomes. Researchers have addressed this by looking at dietary patterns^10 11^ or food types such as whole grains^12^ or red meat.^13^ Navigating this evidence can act as a barrier for researchers not trained in nutrition to interpret this evidence when dietary intervention outcomes are measured in nutrient intake (sugar, salt or fibre). Therefore, it is beneficial to bring together evidence for the health effects of sodium, fats and carbohydrates. Within fats monounsaturated fatty acids (MUFA), polyunsaturated fatty acids (PUFA), saturated fatty acids should be considered independently, as should sugars and fibre within carbohydrates, to identify positive and negative health effects.

Randomised controlled trials provide a robust method to reduce biases, but the duration of follow-up, or sample size, is unlikely to identify a relationship between diet and health events, such as diabetes, cardiovascular disease and cancer. Changes in cardiometabolic measurements for blood pressure, cholesterol and blood glucose can be detected within randomised controlled trials, and can be used as markers for risks of non-communicable diseases to indirectly predict the long-term health impacts. We limited our outcomes to those measure that are typically used in cardiovascular and diabetes risk scores,^14 15^ including blood pressure, cholesterol and HbA1c. Weight was excluded because energy intake was not an exposure of interest.

Despite the large number of systematic reviews collating evidence for individual nutrients, no synthesising evidence for multiple nutrient exposures was found. The aims of this study were to describe the relationships between diet composition described by major nutrient groups and cardiometabolic risk factors. We undertook an umbrella review of reviews to identify estimates from meta-analyses of randomised controlled trials and developed a causal pathways diagram to synthesise the findings.

## Method

The protocol was registered with PROSPERO, CRD42020191611. The design of this umbrella review of reviews^16^ was developed to support public health evaluation of dietary policies.

### Search strategy

Database searches were performed in several databases in Medline, Embase, Cochrane Library and Science Citation Index from 1946 to 26 May 2020. Supplementary searches were conducted of key websites for relevant reports (WHO; Public Health England; Cochrane-hypertension) and reference searching of included reviews.

### Inclusion/exclusion criteria

Studies were included in the review if they assessed fats, fibre, carbohydrate, sugar and salt. We divided the fat category into fatty acids from foods (MUFA, PUFA, saturated fatty acids) and overall fat intake. Studies were included if they measured blood pressure, cholesterol (total, low-density lipid (LDL), or high-density lipid (HDL) or glycaemia (HbA1c). These cardiometabolic outcomes would enable subsequent alignment with epidemiological models for diabetes and cardiovascular risk assessment.^4^ Studies were included into the review if they were a systematic review and meta-analysis of randomised controlled trials or natural experiments with controlled design. Studies were included if they included all adults, or in patients with a relevant metabolic disorder such as diabetes or hypertension.

We excluded studies from observational cohort studies to reduce the risks of bias often identified in nutritional studies.^17^ Children and patients with a health condition other than those identified above were defined as an ineligible population for this review. Individual food products, such as nuts, meat or eggs were excluded to enable the review to focus on the nutrients rather than foods. The aims of the review were to describe effects of nutrient composition, rather than energy intake, on cardiometabolic risks. Given the importance of energy intake for weight gain^18^ and complex system of factors influencing weight gain,^19^ this was excluded as an outcome. Triglycerides were not included in the review because these are not included in the main risk equations under consideration for subsequent modelling work. Fasting plasma glucose was included in the study protocol but was removed during the review because data on effects on HbA1c were more commonly reported.

### Study selection

Studies were screened for inclusion based on the inclusion/exclusion criteria by title and abstract sifting by a reviewer (KS) and 10% were reviewed independently by a second reviewer (PB).

We developed a purposive method of study selection using a sampling framework matrix to stratify the inclusion of evidence by population, exposure (macronutrients) and cardiometabolic risks split by population groups. The method is based on an approach taken to identify evidence for other modelling studies in which a broad scope of evidence is needed.^20^ The method helps to ensure that evidence is represented for all exposures and outcomes and not overwhelmed by the dominant areas of research. The relevant reviews were labelled according to the nutrient components under investigation and cardiometabolic risk factors. This process enabled the reviewers to map the focus of reviews identified, and limit extraction within each category to the most recent evidence available. Studies were selected into the sampling framework matrix by year of publication until two studies were identified for each category, or the list of included studies was exhausted.

The sampling framework matrix was developed to categorise studies by outcome (blood pressure, cholesterol, HbA1c) and nutritional exposure. Nutrient categories were defined as sodium/salt consumption (g), total fat reduction (% total energy intake (TEI)), fatty acids modification from diet, fatty acids modification from supplements, fatty acids modification from both, total carbohydrate reduction (%TEI), fibre (g) and sugars (%TEI). The grouping aimed to identify evidence on substitutions across macronutrient categories (fats and carbohydrate), and also substitutions within these categories, that is, substitution to MUFA from saturated fat.

Experts in nutrition were consulted to review the final study selection and to identity gaps in evidence. Where gaps were identified, additional studies were identified and included to inform these relationships.

### Data extraction

Data on study characteristics were extracted to include review methods, review inclusion criteria (population, study follow-up, study design), summary of geographical locations, number of papers identified and included, number of participants, interventions, controls, planned subgroup analyses and outcomes. All study characteristics were extracted by a single reviewer (KS) with all studies checked (PB, SA, EM).

Data on the mean difference, upper and lower CIs for each exposure and health outcome (systolic or diastolic blood pressure (mm Hg), total cholesterol (mmol/L), HDL cholesterol (mmol/L) and LDL cholesterol (mmol/L), or HbA1c (%)) were extracted separately, including units of measurement. Information on dose sizes, ranges and substitution patterns were extracted. The main study outcomes were extracted unless a subgroup or sensitivity analysis reported exposure from dietary changes, as opposed to capsules or enteral nutrition. Furthermore, exposures in which TEI was not restricted to identify substitution effects were prioritised. Cholesterol effects measured in mg/dL were converted to mmol/L by multiplying by 0.02586. Effects were extracted by a single reviewer (PB) and double checked by two reviewers (SA, EM).

### Quality assessment

All studies included in the study were assessed for quality using the AMSTAR2 checklist.^21^ Quality assessment was undertaken by one reviewer; items that were unclear were discussed. A second reviewer undertook quality assessment of a sample of 10 reviews. We did not exclude any studies on the basis of quality.

### Evidence synthesis and causal pathways diagram

A novel meta-analysis for all causal factors between exposures and health outcomes was not feasible given the large number of exposures and outcomes to be analysed. A narrative synthesis of the data was performed in line with Synthesis without meta-analysis (SWiM) guidance.^22^ Full details of the method of evidence synthesis are described in the online supplemental material. A causal pathways diagram was developed to illustrate findings, to synthesise evidence and depict the links in the nutrient–health relationship. Causal pathway diagrams are useful for summarising and organising information, structure information to validate findings with experts.

10.1136/bmjnph-2023-000666.supp1 Supplementary data

## Results

**Figure 1:**
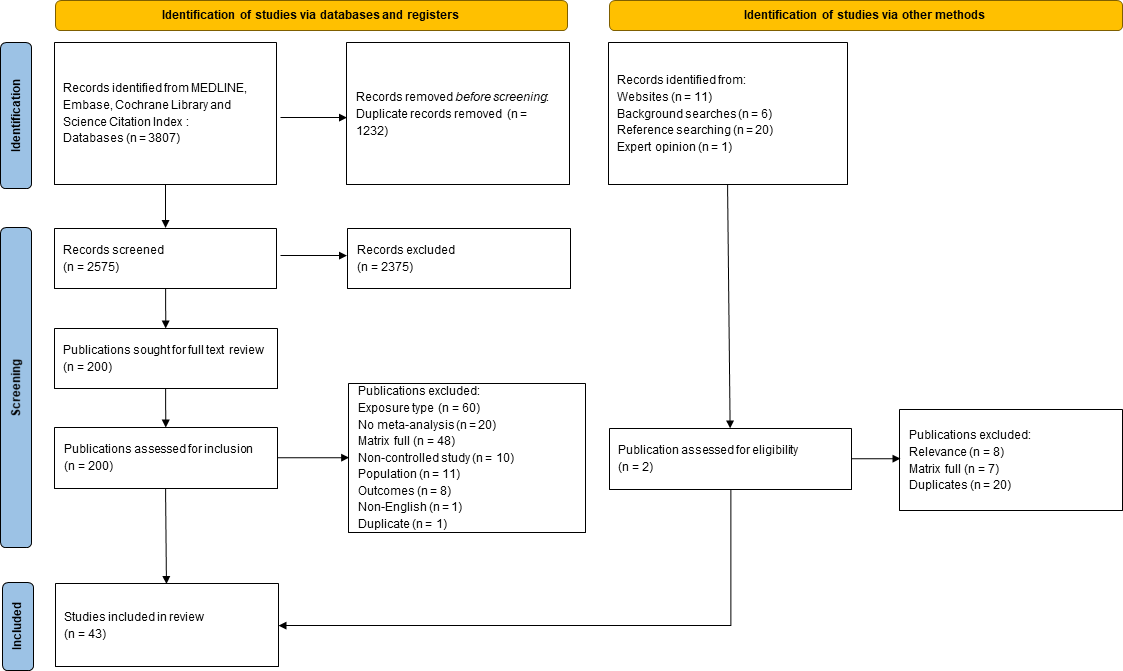
Preferred Reporting Items for Systematic Reviews and Meta-Analyses diagram of selected articles for inclusion in the review.

Database searches identified 2575 and 19 studies were identified in supplementary searches of the grey literature and consultation with nutrition experts. Of these, 43 studies were selected through the process of filling the sampling framework matrix. The full details of the study selection process are detailed in figure 1. An additional study that was used to fill the gap in the review evidence was identified for the impact of substitutions between fatty acids and cholesterol.^23^ The sampling framework matrix of study exposures and outcomes by subpopulation is reported in online supplemental table S1; summary characteristics of the included studies is reported in table 1. During data extraction, an updated version of a Cochrane review was identified.^8^ The outcomes of the AMSTART2 critical appraisal tool assessment for all included studies can be found in online supplemental table S2. Six review studies were assessed as high quality, 4 as moderate quality, 22 as low quality and 11 as critically low.

**Table 1:** Characteristics of systematic reviews examining the effect of nutritional intake on measures of metabolic health in adults

Author	Publication year	Food grouping	Population	Review date	Exposure	No studies	Follow-up restriction	Eligible outcomes
Chewcharat^41^	2020	Fatty acids (Food)	Diabetes	Apr-19	Polyunsaturated fatty acids (Omega-3)	10	None	Blood pressure; cholesterol; HbA1c
Dong^9^	2020	Carbohydrate	Adults	Nov-18	Low carbohydrate diet (<40% TEI)	12	>3 months	Blood pressure; cholesterol
Fechner^28^	2020	Carbohydrate	Adults	Apr-19	Low carbohydrate diet (<45% TEI)	37	None	Blood pressure; cholesterol
Graudal^8^	2020	Sodium	Adults	Apr-16	Sodium	185	None	Blood pressure; cholesterol
Hooper^51^	2020	Fatty acids	Adults	Oct-19	Saturated fatty acids	16	>12 months	Blood pressure; cholesterol
Huang^26^	2020	Sodium	Adults	Jan-19	Sodium	133	None	Blood pressure
Schwingshackl^55^	2020	Sugar	Adults	Aug-18	Dietary sugars and starch	38	None	Cholesterol; HbA1c
Xiao^57^	2020	Fibre	Diabetes	Aug-19	Psyllium consumption	8	None	Cholesterol; HbA1c
Brown^54^	2019	Fatty acids	Adults	Apr-17	Polyunsaturated fatty acids (Omega-3)	83	None	HbA1c
Gjuladin-Hellon^48^	2019	Fat	Adults	Not reported	Carbohydrate restricted diets; low-fat diets	8	>6 months	Cholesterol
Javonavski^37^	2019	Fatty acids	Adults	Jun-17	Monounsaturated fatty acids	35	>3 week	Blood pressure
Jovanovski^60^	2019	Fibre	Diabetes	Jun-18	Viscous fibre supplementation	28	>3 weeks	HbA1c
Korsmo-Haugen^30^	2019	Carbohydrate	Diabetes	Jan-16	Low carbohydrate (<40% TEI)	23	>3 months	Cholesterol; HbA1c
McArdle^68^	2019	Carbohydrate	Diabetes	Apr-19	Low carbohydrate diet	25	>8 weeks	Blood pressure; cholesterol; HbA1c
Natto^52^	2019	Fatty acids	Diabetes	Jan-18	Polyunsaturated fatty acids (Omega-3)	16	None	Cholesterol HbA1c
Neuenschwander^50^	2019	Carbohydrate Fat	Diabetes	Jan-18	Low carbohydrate diet; low-fat diet	52	>3 months	Cholesterol
Pan^49^	2019	Carbohydrate Fat	Diabetes	Dec-16	Low carbohydrate diet; low-fat diet	10	None	Cholesterol; HbA1c
Reynolds^32^	2019	Fibre	Adults	Feb-18	Total dietary fibre	185	>4 weeks	Blood pressure; cholesterol; HbA1c
Schwingshackl^27^	2019	Carbohydrate Fat	Adults	Jun-17	Low carbohydrate; low sodium diet; low-fat diet	67	None	Blood pressure
Abelhamid^39^	2018	Fatty acids	Adults	Apr-17	Polyunsaturated fatty acids	183	>12 months	Blood pressure; cholesterol
Hooper^40^	2018	Fatty acids	Adults	May-17	Polyunsaturated fatty acids (Omega-6)	19	>12 months	Cholesterol
Huntriss^31^	2018	Carbohydrate	Diabetes	Jun-16	Low carbohydrate diet	18	>48 weeks	Blood pressure; cholesterol; HbA1c
Khan^33^	2018	Fibre	Adults	Jun-17	Dietary or supplementary fibre	22	>4 weeks	Blood pressure
Lu^47^	2018	Fat	Adults	Oct-16	Low-fat diet	20	None	Blood pressure; cholesterol
Noronha^61^	2018	Sugar	Adults	Apr-18	Dietary sugars	14	None	HbA1c
O’Mahoney^42^	2018	Fatty acids	Diabetes	Jul-17	Polyunsaturated fatty acids (Omega-3)	45	None	Blood pressure; cholesterol; HbA1c
Schwingschakl^58^	2018	Fat; carbohydrate	Diabetes	Jul-17	Low-fat diet; low carbohydrate diet	56	>12 weeks	HbA1c
Hartley^34^	2016	Fibre	Adults	Jan-15	Dietary fibre	23	None	Blood pressure; cholesterol
Imamura^59^	2016	Fatty acids	Adults	Nov-15	Saturated fatty acids; monounsaturated fatty acids; polyunsaturated fatty acids; carbohydrates	102	>4 week	HbA1c
Mensink	2016	Fatty acids	Adults	Dec-13	Saturated fatty acid intake	84	None	Cholesterol
Qian^36^	2016	Fatty acids	Diabetes	Mar-15	Monounsaturated fatty acids	28	>2 weeks	Blood pressure; cholesterol HbA1c
Miller^38^	2014	Fatty acids	Hypertension	Feb-13	Polyunsaturated fatty acids (Omega-3)	70	None	Blood pressure
Te Morenga^44^	2014	Sugar	Adults	Aug-13	Sucrose or free sugars	13	>2 weeks	Cholesterol; blood pressure
Bueno^56^	2013	Carbohydrate	Adults	Aug-12	Low carbohydrate (<50 g or <10% TEI)	14	>12 months	Blood pressure; cholesterol; HbA1c
He^25^	2013	Sodium	Adults	Nov-12	Reduction in urinary sodium	34	>4 weeks	Blood pressure; cholesterol
Cozma^62^	2012	Sugar	Diabetes	Mar-12	Fructose	16	>1 week	HbA1c
Ha^35^	2012	Sugar	Adults	Jan-12	Fructose	15	>1 week	Blood pressure
Hooper^45^	2012	Fat/carbohydrate	Adults	Jun-10	Low-fat diet; low carbohydrate diet	48	>6 months	Blood pressure; cholesterol
Hu^46^	2012	Fat/carbohydrate	Adults	Jun-11	Low-fat diet; low carbohydrate diet	23	>6 months	Blood pressure; cholesterol
Santos^29^	2012	Carbohydrate	Adults	Mar-11	Low carbohydrate diet (defined by author)	19	>3 months	Blood pressure; cholesterol; HbA1c
WHO^24^	2012	Sodium	Adults	Aug-11	Sodium	37	>4 weeks	Blood pressure; cholesterol
Sievenpiper^53^	2009	Sugar	Diabetes	Feb-09	Fructose	16	>1 week	Cholesterol
Hartweg^43^	2007	Fatty acids	Diabetes	Feb-06	Polyunsaturated fatty acids (Omega-3)	34	None	Blood pressure

### Blood pressure

We found that sodium increased systolic blood pressure (overall range: −3.39 mm Hg to −4.26 mm Hg) and diastolic blood pressure (overall range: −1.54 mm Hg to −2.07 mm Hg) and the estimates were statistically significant.^24–26^ The effects on blood pressure were larger for a hypertensive population (overall range: −1.50 mm Hg to −7.83 mm Hg) compared with normotensive populations (overall range: −0.66 mm Hg to −7.75 mm Hg).^8 24–27^

Low carbohydrates diet decreased systolic and diastolic blood pressure^9 27–31^ and the results were significant in some studies and subgroup analyses.^9 28 29 31^ There was evidence to suggest that increased fibre is associated with a reduction in systolic blood pressure (overall range: −1.59 to −1.27 mm Hg), and diastolic blood pressure (overall range: −2.40 to −0.39 mm Hg),^32–34^ and the associations were statistically significant in most studies.^32 33^ One study found that replacing carbohydrate with fructose decreased diastolic blood pressure.^35^

In individuals with diabetes, replacing carbohydrates with MUFA significantly reduced systolic blood pressure (mean: −2.31 mm Hg),^36^ but not in general populations.^37^ PUFA were not statistically significantly associated with lower systolic blood pressure and diastolic blood pressure in general populations^38–40^ or diabetes populations.^41–43^ There was no evidence for a significant relationship between low-fat diets, or sugars and systolic blood pressure.^35 44^

### Cholesterol

Sodium was associated with an increase in total cholesterol (overall range: 0.02–0.13 mmol/L).^8 24 25^ The relationship was statistically significant in the most recent evidence review.^8^ Low-fat diets substituting fats for carbohydrate were found to reduce total cholesterol (overall range: −0.01 to −0.09 mmol/L).^45–50^ The difference was statistically significant in two out of five studies.^45 46^ Increasing MUFA to replace saturated fat was significantly associated with a reduction in total cholesterol (mean: −0.05 mmol/L).^23^ Increasing PUFA to replace saturated fat, monounsaturated fat or other dietary energy was associated with lower total cholesterol (overall range: −0.06 to −0.33 mmol/L) in the general population, and the relationships were statistically significant.^23 39 40 51^ Two studies in patients with diabetes were not statistically significant.^41 52^ In general populations increasing saturated fat to replace either carbohydrate^23 51^ or any foods^51^ was found to increase total cholesterol (overall range: 0.05–0.24 mmol/L) and the findings were statistically significant.^23 51^

There was evidence that low carbohydrate diets increased total cholesterol (overall range: 0.07–0.13 mmol/L) in the general population, and some estimates were statistically significant,^9 28 46^ but not statistically significant in diabetes populations.^30 31 49 50^ There is evidence for a relationship between fibre and total (overall range: −0.15 to −0.21) and the association was statistically significant for total cholesterol in one study.^32^ There is evidence to suggest that dietary-free sugars significantly increase total cholesterol (mean: 0.23 mmol/L),^44^ but not in patients with diabetes.^53^

In general populations, low-fat diets substituting fat for carbohydrate reduced HDL cholesterol (overall range: −0.01 to −0.09 mmol/L),^45–48^ and the relationship was significant^46–48^ or borderline significant.^45^ Increasing MUFA to replace saturated fat was significantly associated with lower HDL cholesterol (mean: −0.002 mmol/L).^23^ One study identified a statistically significant relationship between PUFA replacing saturated fat and lower HDL cholesterol (mean:−0.005 mmol/L),^23^ whereas three reported non-significant findings.^39 40 54^ Two studies of PUFA replacing other dietary energy in populations with diabetes report different direction of effects for HDL^41 52^ and both were statistically significant. In general populations, increasing saturated fat to replace carbohydrate or any foods was found to significantly increase HDL cholesterol (overall range: 0.01–0.011 mmol/L) and the findings were statistically significant.^23 51^

There was evidence that low carbohydrate diets increased HDL cholesterol (overall range: 0.04–0.10 mmol/L)^9 28–31 46 49 50^ and the relationships were statistically significant in some studies or subanalyses.^9 28 29 31 46^ Dietary-free sugars significantly increased HDL cholesterol (mean: 0.02 mmol/L).^44^ In a general population, substitution between sucrose, fructose, starch and glucose was not statistically significant.^55^ There was no evidence of a statistically significant effect for either sodium or fibre on HDL cholesterol.

In general populations, low-fat diets substituting fat for carbohydrate reduced LDL cholesterol (overall range: −0.01 to −0.11 mmol/L),^45–48^ and the relationship was significant in two studies.^45 46^ Increasing MUFA to replace saturated fat was significantly associated with lower LDL cholesterol (mean: −0.04).^23^ We found statistically significant effects for PUFA to replace saturated fat on LDL cholesterol (overall range: −0.04 to −0.48),^23 51^ but not when replacing other dietary energy.^39 40^ Three studies of PUFA in populations with diabetes reported non-significant findings.^36 41 52^ In general populations, increasing saturated fat to replace carbohydrate, or any foods, was found to significantly increase LDL cholesterol (overall range: 0.03–0.19 mmol/L) and the findings were statistically significant in the majority of analyses.^23 51^

There was evidence that low carbohydrate diets increased LDL cholesterol (overall range: 0.10–0.11 mmol/L)^9 28 29 46 50 56^ and the relationships were statistically significant in some studies or analyses.^9 28 46 50 56^ There is evidence for a relationship between fibre and LDL cholesterol (overall range: −0.10 to −0.23).^32 34 57^ There is evidence to suggest that dietary-free sugars significantly increase LDL cholesterol (mean: 0.17 mmol/L).^44^ Substitution from starch to sucrose or glucose increases LDL cholesterol^55^ but not fructose.^53^ There were no statistically significant effects for sodium on LDL cholesterol.

### Glycaemia (HbA1c)

In populations with diabetes, there was evidence that low-fat diets substituting for carbohydrates decrease HbA1c (overall range: −0.17% to −0.47%) and was statistically significant in one study,^58^ but not statistically significant in another.^49^

Increasing MUFA was associated with a significant reduction in HbA1c when substituted for carbohydrate or saturated fat (overall range: −0.09% to −0.12%) for the general population^59^ and non-statistically significant in a population with diabetes when substituted for carbohydrate.^36^ Increasing PUFA to replace carbohydrate or saturated fat was associated with a decrease in HbA1c (overall range: −0.02% to −0.33%),^41 42 52 54 59^ and the relationships were statistically significant in one study.^59^

There is evidence for fibre consumption decreasing HbA1c in populations with diabetes (overall range: −0.61 to −0.91) and the finding was statistically significant.^57 60^ The association was not statistically significant in a general population.^32^

There is evidence to suggest that fructose and tagatose are associated with a decrease in HbA1c in general populations^61^ and populations with diabetes.^62^ Substitutions between fructose, sucrose, glucose and starch were not associated with significant changes to HbA1c.^55^ There was no statistically significant effect of low sodium diet on HbA1c.

### Summary data and causal pathway diagram

**Figure 2:**
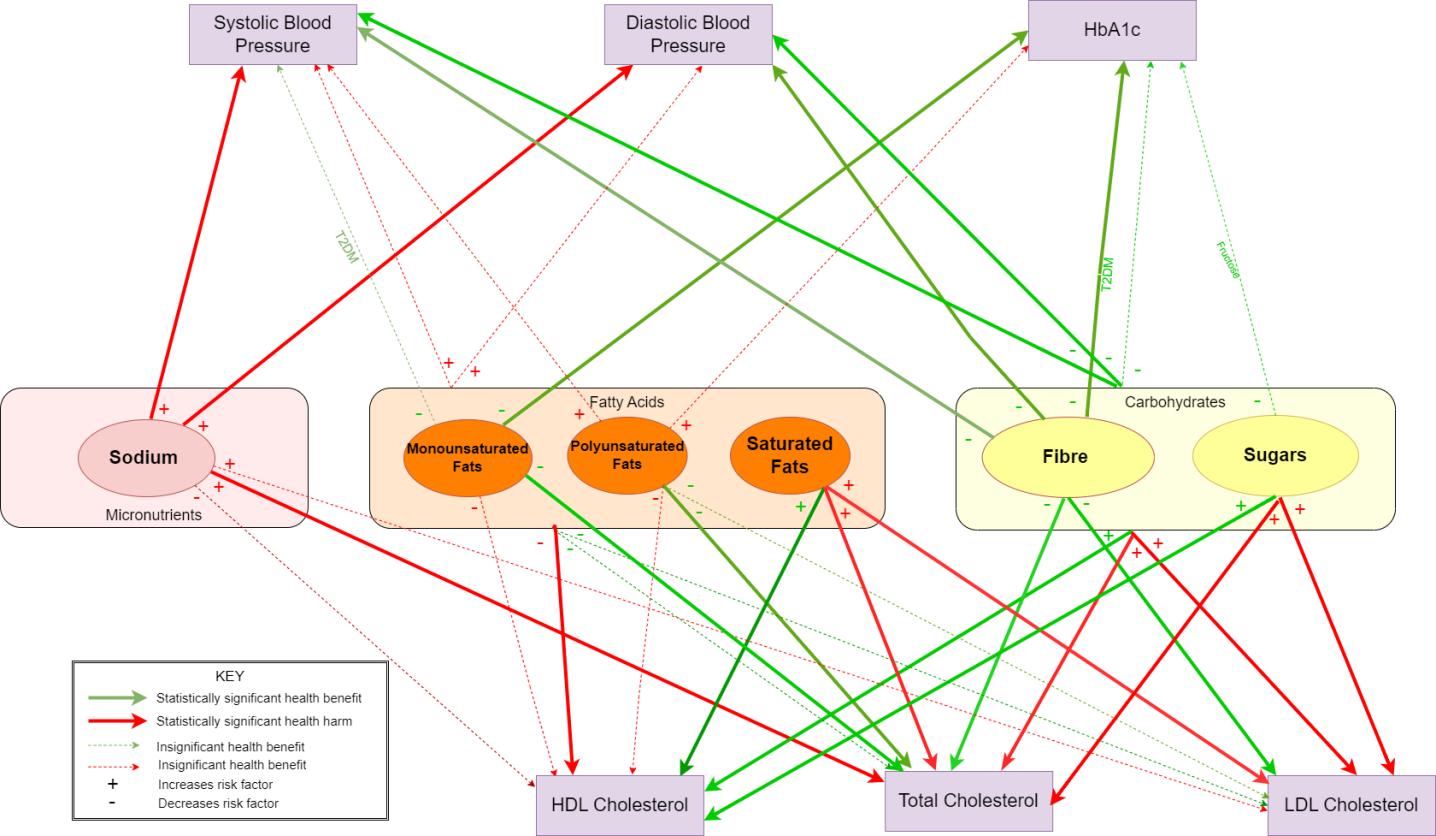
A causal pathway diagram illustrating the direction, and strength of evidence between nutrients and metabolic markers. HDL, high-density lipid; LDL, low-density lipid.

A summary of effects size and significance for relationships for the general population is provided in table 2, and individual study effects are reported in the online supplemental tables. Figure 2 illustrates the evidence in a causal pathway diagram to illustrate the evidence.

**Table 2:** Description of the direction, statistical significance and certainty of reported relationships between nutrients and metabolic risks for the general population, unless otherwise indicated

Exposure	Outcome	Direction of effect (range)	Statistically significant	Certainty of evidence	Number of systematic reviews	Number of RCT studies in meta-analysis	Subgroup heterogeneity
Sodium	Systolic blood pressure	−3.39 to −4.26 mm Hg	Yes	High	3	34–135	Yes
Sodium	Diastolic blood pressure	−1.54 to −2.07 mm Hg	Yes	High	3	34–135	Yes
Sodium	Total cholesterol	0.02 to −0.13 mmol/L	Yes	Low	3	8–28	No
Sodium	HDL cholesterol	−0.01 to −0.02 mmol/L	No	High	3	6–20	No
Sodium	LDL cholesterol	0.03 to 0.06 mmol/L	No	High	3	5–18	No
All fat	Systolic blood pressure	−0.56 to 1.55 mm Hg	No	Low	3	6–18	No
All fat	Diastolic blood pressure	−0.25 to 2.18 mm Hg	No	Low	3	6–18	No
All fat	Total cholesterol	−0.18 to −0.01 mmol/L	No	Low	4	15–16	No
All fat	HDL cholesterol	−0.09 to −0.01 mmol/L	Yes	Low	4	15–19	Yes
All fat	LDL cholesterol	−0.11 to −0.01 mmol/L	No	Low	4	14–19	No
All fat	HbA1c (diabetes only)	−0.47% to −0.17%	No	Low	2	2–10	No
MUFA	Systolic blood pressure	−0.08 mm Hg	No	Low	1	14	Yes
PUFA	Systolic blood pressure	−1.52 to −0.47 mm Hg	No	Low	3	2–93	No
Saturated fat	Systolic blood pressure	−0.19 mm Hg	No	High	1	5	No
MUFA	Diastolic blood pressure	0.01 mm Hg	No	Low	1	14	No
PUFA	Diastolic blood pressure	−0.99 to 0.24 mm Hg	No	Low	3	2–92	No
Saturated fat	Diastolic blood pressure	−0.39 mm Hg	No	High	1	5	No
MUFA	Total cholesterol	−0.05 mmol/L	Yes	Low	1	74	No
PUFA	Total cholesterol	−0.33 to −0.05 mmol/L	Yes	High	2	2–74	Yes
Saturated fat	Total cholesterol	0.05–0.24 mmol/L	Yes	Low	2	14	No
MUFA	HDL cholesterol	−0.002 mmol/L	Yes	Low	1	68	Yes
PUFA	HDL cholesterol	−0.0 to 0.00 mmol/L	No	Low	4	18–68	No
Saturated fat	HDL cholesterol	0.01–0.011 mmol/L	Yes	High	2	6	No
MUFA	LDL cholesterol	−0.04 mmol/L	Yes	Low	1	69	Yes
PUFA	LDL cholesterol	−0.48 to −0.04 mmol/L	No	Low	4	15–69	No
Saturated fat	LDL cholesterol	0.03–0.16 mmol/L	Yes	High	2	5–69	No
MUFA	HbA1c	−0.12% to −0.09%	Yes	Low	1	23	No
PUFA	HbA1c	−0.11% to −0.02%	Yes	Low	2	16–23	No
Saturated fat	HbA1c	0.03%	No	Low	1	23	No
All carbohydrates	Systolic blood pressure	−4.81 to −1.10 mm Hg	No	Low	4	18–24	No
All carbohydrates	Diastolic blood pressure	−3.10 to −1.07 mm Hg	No	Low	4	18–24	No
All carbohydrate	Total cholesterol	0.07–0.13 mmol/L	Yes	Low	3	14–31	No
All carbohydrate	HDL cholesterol	0.04–0.10 mmol/L	Yes	Low	3	19–37	No
All carbohydrate	LDL cholesterol	−0.07 to 0.11 mmol/L	Yes	Low	4	19–37	No
All carbohydrate	HbA1c	−0.21%	No	Low	1	6	No
Fibre	Systolic blood pressure	−1.59 to −1.27 mm Hg	Yes	Low	3	4–22	Yes
Fibre	Diastolic blood pressure	−2.40 to −0.39 mm Hg	Yes	Low	3	4–22	Yes
Fibre	Total cholesterol	−0.16 to −0.15 mmol/L	Yes	Low	2	7–36	No
Fibre	HDL cholesterol	−0.03 to 0.01 mmol/L	No	High	2	6–32	No
Fibre	LDL cholesterol	−0.14 to −0.10 mmol/L	Yes	High	2	7–34	No
Fibre	HbA1c	−0.35%	No	High	1	6	Yes
Free sugar	Systolic blood pressure	−0.24 mm Hg	No	Low	1	12	No
Free sugar	Diastolic blood pressure	0.65 mm Hg	No	Low	1	12	No
Free sugar	Total cholesterol	0.23 mmol/L	Yes	Low	1	36	No
Free sugar	HDL cholesterol	0.02 mmol/L	Yes	Low	1	29	No
Free sugar	LDL cholesterol	0.17 mmol/L	Yes	Low	1	22	No
Fructose	Systolic blood pressure	−1.10 mm Hg	No	Low	1	11	No
Fructose	Diastolic blood pressure	−1.54 mm Hg	Yes	Low	1	11	No
Fructose	LDL cholesterol	0.22 mmol/L	Yes	Low	1	38	No
Fructose	HbA1c	−0.38% to 0.29%	Yes	Low	2	7–38	No

HDL, high-density lipid; LDL, low-density lipid; MUFA, monounsaturated fatty acids; PUFA, polyunsaturated fatty acids.

## Discussion/conclusion

### Main findings of this study

The review serves the function of mapping the nutrient exposures and cardiometabolic outcomes. It has identified evidence across nutrients, cardiometabolic risk factors and considered variations in effects across population subgroups. The findings are illustrated in a causal pathway diagram. The review summarises current understanding of the non-weight relationships between dietary quality and cardiometabolic risks, and provides researchers with a resource to justify the health benefits of dietary change. The review has highlighted the harms of sodium on blood pressure, particularly in those with hypertension. Whereas fibre and unsaturated fats can reduce systolic blood pressure. The relationships between fats and carbohydrates on cholesterol vary by the types of macronutrients, so that fibre and starch decrease cholesterol, whereas sugar and saturated fat increase cholesterol. MUFA, sugar and fibre were associated with HbA1c. Many of the studies included in the review were found to be a low grade of evidence. There were many cases where the findings from reviews with similar exposures and outcomes were conflicting. This may be due to the differences in study objectives and inclusion criteria but may also be impacted by changes in evidence over time. As such, the findings should be interpreted with caution. In synthesising the evidence, we considered the quality of studies, but have not excluded the findings from low-quality studies. Further research could update formal synthesis of the nutrients and cardiometabolic risks using consistent methods.

### What is already known on this topic?

The direction of relationships between macronutrients and cardiometabolic risks are consistent with national^63^ and international guidelines^64^ to restrict the consumption of salt, saturated fat and increase consumption of fruit and vegetables to increase dietary fibre. We only identified a significant relationship between free sugars and cholesterol, and none for a relationship between sugar and HbA1c. This finding that there are few studies identifying significant effects of sugar on cardiometabolic risks is consistent with other reviews of the relationship between carbohydrate and health.^65^ However, given the reviews exclusion of weight gain as a measure of metabolic health, the negative health effects of free sugars diet may not be fully represented within the scope of this review.

### What this study adds

This umbrella review of reviews provides a comprehensive search and mapping of the literature. The findings have been combined in a narrative synthesis, and causal pathway diagram to indicate the effects of various macronutrient components based on the most recent available evidence. The purposive sampling of studies through a sampling framework matrix enabled the reviewers to identify evidence from a range of dietary macronutritional components across various population groups, also identifying gaps and uncertainty in the evidence.

The study has highlighted gaps and uncertainty in the evidence for associations between nutrients and cardiometabolic risks. Few studies have investigated the association between sugar and cardiometabolic risks. We note that despite the large number of studies investigating the relationships between sodium and blood pressure, none have reported associations with HbA1c. Recent findings from observational studies highlight a relationship between sodium and HbA1c in a non-hypertensive population.^66^ There is a high degree of uncertainty in the evidence identified in this review, with inconsistent and conflicting evidence across many of the relationships we have reviewed.

### Limitations of this study

A limitation of the study is that the reviews were not statistically combined in favour of a narrative assessment of outcomes and strength of evidence. The inclusion of all relevant reviews in this field would either contain dietary interventions too heterogenous to be combined statistically or would not add to the findings from the reviews. The review does not illustrate dietary impacts on triglycerides, or other measures of glycaemia that may be of interest to nutritionists, epidemiologists and other health professionals, because these are not commonly used in assessing cardiometabolic risk. It was necessary to prioritise certain dietary changes and metabolic risks for this review, but further research could extend this approach to accommodate evidence on single micronutrients that have been associated with reductions in blood pressure.^67^

Purposive sampling may have excluded important studies and evidence that may strengthen or conflict with the summaries provided here. However, selecting more recent systematic reviews should capture the most contemporary evidence.

## Data Availability

No data are available. All data used in the study have been obtained from published sources.
