# Supplementary material for: Relationships between sodium, fats and carbohydrates on blood pressure, cholesterol and HbA1c: an umbrella review of systematic reviews
Source: BMJ Nutr Prev Health. 2024 Mar 21;7(1):191–203. doi: 10.1136/bmjnph-2023-000666 (PMC11221289; doi:10.1136/bmjnph-2023-000666)
Supplement: Supplementary data [file bmjnph-2023-000666supp001.pdf]

BMJ Publishing Group Limited (BMJ) disclaims all liability and responsibility arising from any reliance placed on this supplemental material which has been supplied by the author(s)

Search Strategy

Database: Ovid MEDLINE(R) and Epub Ahead of Print, In-Process & Other Non-Indexed Citations and Daily <1946 to May 22, 2020>

Search Strategy:

----

1 "systolic blood pressure".ab,ti. (52770)
2 total cholesterol.ab,ti. (50412)
3 cholesterol, hdl/ or cholesterol, ldl/ (42093)
4 ((hdl or ldl) adj cholesterol).ab,ti. (41766)
5 Glycated Hemoglobin A/ (34591)
6 hba1c.ab,ti. (33122)
7 "fasting plasma glucose".ab,ti. (12429)
8 dietary carbohydrates/ or dietary fiber/ or dietary sugars/ or dietary sucrose/ or high fructose corn syrup/ or starch/ (60407)
9 (carbohydrate$ or glucose$ or starch$ or sucrose$ or lactose$ or fiber$).ab,ti. (922668)
10 dietary fats/ or cholesterol, dietary/ or dietary fats, unsaturated/ (58784)
11 ("transfatty acid$" or "saturated fat$" or "mono-unsaturated fat$" or "poly-unsaturated fat$").ab,ti. (18019)
12 salt.ab,ti. (140846)
13 sodium.ab,ti. (345743)
14 (tfa or "trans-fatty acid$" or "monounsaturated fat$").ab,ti. (10238)
15 fatty acids/ or trans fatty acids/ (85646)
16 (polyunsaturated fat$ or pufa).ab,ti. (29925)
17 8 or 9 or 10 or 11 or 12 or 13 or 14 or 15 or 16 (1497420)
18 MEDLINE.tw. (116087)
19 systematic review.tw. (153736)
20 meta analysis.pt. (114984)
21 18 or 19 or 20 (275238)
22 1 or 2 or 3 or 4 or 5 or 6 or 7 (198420)
23 17 and 22 (66317)
24 21 and 23 (1738)
25 limit 24 to english language (1705)
26 limit 24 to humans (1434)
27 from 26 keep 1-1434 (1434)

Table S1: Sampling framework matrix to stratify the inclusion of evidence by population, exposure and health outcome

		All Fat	Fatty Acids (Food)	Fatty Acids (Food and supplement)	All carbohydrate	Fibre	Sugars	Sodium/Salt
General Population	HbA1c		Brown 2019 (40) Imamura 2016 (39)	Brown 2019 (40)	Bueno 2013 (46) Santos 2012 (43)	Reynolds 2019 (48)	Schwingshackl 2020 (55) Noronha 2018 (56)	
	Blood Pressure	Lu 2018 (24) Hooper 2012 (22) Hu 2012 (23)	Jovanovski 2019 (29) Abelhamid 2018 (32) Hooper 2018 (33)	Abelhamid 2018 (32) Hooper 2018 (33)	Dong 2020 (42) Fechner 2020 (41)	Reynolds 2019 (48) Khan 2018 (49)	Te Morenga 2014 (53) Ha 2012 (54)	Huang 2020 (20)
	Cholesterol	Lu 2018 (24) Gjuladin-Hellon 2019 (25)	Hooper 2020 (38) Brown 2019 (40)	Abelhamid 2018 (32) Hooper 2018 (33)	Dong 2020 (42) Fechner 2020 (41)	Reynolds 2019 (48) Hartley 2016 (50)	Schwingshackl 2020 (55) Te Morenga 2014 (53)	Graudal 2020 (17) WHO 2012 (18)
Diabetes	HbA1c	Pan 2019 (26) Schwingschakl 2018 (28)	Chewcharat 2020 (34) Qian 2016 (30)	Natto 2019 (37) O'Mahoney 2018 (35)	Korsmo-Haugen 2019 (44) McArdle 2019 (47) Pan 2019 (26)	Jovanovski 2019 (52) Xiao 2020 (51)	Cozma 2012 (57)	
	Blood Pressure		Qian 2016 (30) Hooper 2018 (33)	O'Mahoney 2018 (35) Hartweg 2007 (36)	Korsmo-Haugen 2019 (44) Huntriss 2018 (45)		Ha 2012 (54)	
	Cholesterol	Neuenschwander 2019 (27) Pan 2019 (26)	Chewcharat 2020 (34) Qian 2016 (30)	Natto 2019 (37) O'Mahoney 2018 (35)	Korsmo-Haugen 2019 (44) Neuenschwander 2019 (27) Pan 2019 (26)	Xiao 2020 (51)	Sievenpiper 2009 (63)	
Hypertension	HbA1c							
	Blood Pressure	Schwingshackl 2019 (21)	Miller 2014 (31)	Miller 2014 (31)	Schwingshackl 2019 (21)	Khan 2018 (49)		Schwingshackl 2019 (21) Graudal 2020 (17)
	Cholesterol							Graudal 2020 (17) He 2013 (19)

Table S2: AMSTAR 2 quality assessment checklist and outcome for included systematic reviews

	1. Did the research questions and inclusion criteria for the review include the components of PICO?	2. Did the report of the review contain an explicit statement: the review methods were established prior to the conduct of the review and did the report justify any significant deviations from the protocol?	3. Did the review authors explain their selection of the study designs for inclusion in the review?	4. Did the review authors use a comprehensive literature search strategy?	5. Did the review authors perform study selection in duplicate?	6. Did the review authors perform data extraction in duplicate?	7. Did the review authors provide a list of excluded studies and justify the exclusions?	8. Did the review authors describe the included studies in adequate detail?	9. Did the review authors use a satisfactory technique for assessing the risk of bias (RoB) in individual studies that were included in the review?	10. Did the review authors report on the sources of funding for the studies included in the review?	11. If meta-analysis was performed did the review authors use appropriate methods for statistical combination of results?	12. If meta-analysis was performed, did the review authors assess the potential impact of RoB in individual studies on the results of the meta-analysis or other evidence synthesis?	13. Did the review authors account for RoB in individual studies when interpreting/discussing the results of the review?	14. Did the review authors provide a satisfactory explanation for, and discussion of, any heterogeneity observed in the results of the review?	15. If they performed quantitative synthesis did the review authors carry out an adequate investigation of publication bias and discuss its likely impact on the results of the review?	16. Did the review authors report any potential sources of conflict of interest, including any funding they received for conducting the review?	Assessment
Abelhamid 2018	Y	Y	N	Y	N	Y	Y	Y	Y	Y	Y	Y	Y	y	Y	Y	Low
Brown, 2019	Y	Y	N	PY	y	Y	N	y	Y	N	Y	Y	Y	Y	Y	Y	Low
Bueno 2013	Y	Y	Y	Y	Y	Y	PY	PY	Y	N	Y	Y	Y	Y	Y	Y	High
Chewcharat, 2020	Y	Y	Y	PY	Y	N	N	Y	Y	N	Y	Y	Y	y	Y	Y	Low
Cozma 2013	Y	N	Y	PY	N	N	Y	N	N	Y	Y	N	Y	Y	Y	Y	Critically low
Dong, 2020	Y	N	N	PY	N	N	Y	Y	Y	Y	Y	Y	Y	Y	Y	Y	Low
Fechner, 2020	Y	N	N	PY	Y	N	N	Y	N	N	Y	Y	Y	Y	Y	Y	Critically low
Gjuladin-Hellon, 2019	Y	N	N	PY	N	N	Y	Y	Y	N	Y	Y	Y	Y	Y	Y	Low
Graudal 2020	Y	Y		Y	Y	Y	Y	Y	Y	Y	Y	Y	Y	y	Y	Y	High
Ha 2012	Y	N	N	N	N	Y	N	N	N	Y	Y	Y	Y	Y	Y	Y	Critically low
Hartley 2016	Y	Y	Y	PY	Y	Y	Y	Y	Y	Y	Y	Y	Y	Y	Y	Y	High
Hartweg 2007b	Y	PY	N	PY	NR	NR	NR	Y	Y	N	Y	Y	PY	Y	Y	Y	Low
He 2013	Y	N	N	PY	N	Y	N	Y	Y	N	Y	Y	Y	Y	Y	Y	Critically low
Hooper 2012	Y	PY	Y	Y	Y	Y	Y	y	Y	N	Y	Y	Y	Y	Y	Y	High
Hooper, 2018	Y	Y	N	Y	N	Y	Y	Y	Y	Y	Y	Y	Y	y	Y	Y	Low
Hooper 2020	Y	Y	Y	Y	Y	Y	Y	Y	Y	Y	Y	Y	Y	Y	Y	Y	High
Hu 2012	Y	Y	Y	PY	y	y	N	Y	PY	N	Y	Y	PY	Y	Y	Y	Low
Huang, 2020	Y	Y	N	PY	Y	Y	N	Y	Y	N	Y	Y	Y	y	Y	Y	Low
Huntriss, 2018	Y	Y	N	PY	N	N	N	Y	Y	N	Y	N	Y	y	N	Y	Critically low
Imamura 2016	Y	Y	Y	Y	Y	Y	N	Y	Y	N	Y	Y	Y	y	Y	Y	Low
Janovanski, Castro 2019	Y	PY	N	Y	N	Y	N	Y	Y	Y	Y	Y	Y	Y	Y	Y	Low
Jovanovski,, Khayyat,2019	Y	Y	N	PY	Y	y	N	Y	y	Y	Y	Y	Y	Y	Y	Y	Low
Khan 2018	Y	Y	N	PY	N	Y	N	Y	Y	Y	Y	Y	Y	Y	Y	Y	Low
Korsmo-Haugen, 2019	Y	Y	N	PY	Y	Y	Y	Y	Y	N	Y	Y	Y	Y	Y	Y	Moderate
Lu, 2018	Y	N	N	PY	N	N	N	PY	Y	N	Y	Y	Y	y	Y	N	Critically low
McArdle, 2019	Y	Y	N	PY	Y	Y	N	Y	Y	N	Y	N	Y	y	Y	Y	Low
Mensink	Y	N	Y	N	N	N	N	Y	Y	Y	Y	Y	Y	N	N	N	Critically low
Miller 2014	Y	N	N	PY	NR	N	N	Y	N	N	Y	Y	Y	Y	Y	Y	Critically low
Natto, 2019	Y	y	N	PY	y	y	N	Y	Y	N	Y	N	Y	y	Y	Y	Low
Neuenschwander 2019	Y	Y	N	PY	Y	Y	Y	Y	Y	N	Y	Y	Y	y	Y	Y	Moderate
Noroha, 2018	Y	PY	N	PY	N	Y	N	Y	Y	Y	Y	Y	Y	y	Y	Y	Low
O'Mahoney 2018	Y	Y	Y	Y	Y	Y	N	Y	Y	N	Y	Y	Y	Y	Y	Y	Low
Pan, 2019	Y	Y	N	PY	Y	Y	N	PY	Y	N	Y	Y	Y	y	N	N	Critically low
Quian 2016	Y	N	N	PY	y	y	N	Y	Y	N	Y	Y	Y	Y	Y	Y	Low
Reynolds 2019	Y	Y	Y	Y	Y	Y	NR	Y	Y	Y	Y	Y	Y	Y	Y	Y	High
Santos 2012	Y	N	Y	PY	Y	N	PY	Y	N	N	Y	N	N	N	N	Y	Critically low
Schwingschakl 2018	Y	Y	Y	PY	Y	Y	Y	Y	Y	N	Y	Y	Y	Y	Y	Y	High
Schwingshackl, 2019	Y	Y	N	PY	Y	Y	Y	Y	Y	N	Y	Y	Y	y	Y	Y	Moderate
Schwingshackl, 2020	Y	Y	N	PY	N	Y	y	Y	Y	Y	Y	Y	Y	y	Y	Y	Moderate
Sievenpiper 2009	Y	N	N	y	N	Y	N	Y	N	N	Y	Y	Y	Y	Y	y	Critically Low
Te Morenga 2014	y	N	y	y	y	y	y	y	y	Y	Y	Y	Y	Y	Y	Y	Low
WHO 2012	Y	N	Y	Y	Y	Y	Y	Y	Y	Y	Y	Y	Y	Y	Y	Y	Low
Xiao 2020	Y	N	N	PY	Y	Y	N	Y	Y	N	Y	Y	Y	Y	Y	Y	Critically low

Table S3: The impact of sodium changes on systolic blood pressure

Description of the population/group	Description of change in exposure	Change in Systolic Blood Pressure (mmHg)	Clinically meaningful difference	Reference
General Population	Reduced sodium intake vs. usual sodium intake.	-3.39 (-4.31, -2.46)	Yes	WHO 2012
	Reduced sodium intake (-4.4g/day) vs. usual sodium intake	−4.18 (−5.18,−3.18)	Yes	He 2013
	Reduced sodium intake vs. usual sodium intake	-4.26 (−4.89, −3.62)	Yes	Huang 2020
Hypertensives	Reduced sodium intake vs. usual intake	–4.06 (–5.15, –2.96)	Yes	WHO 2012
	Reduced sodium intake (-4.4g/day) vs. usual intake	−5.39 (−6.62, −4.15)	Yes	He 2013
	Reduced sodium intake	-2.76 (-3.22 to -2.31)	Yes	Huang 2020
	Low Sodium diet	-2.96 (-4.74, -1.71)	Yes	Schwingschakl 2019
Hypertensives (White)	Low sodium intake vs. high sodium intake	-5.71 [-6.67 to -4.74]	Yes	Graudal 2020
	Reduced sodium intake)-4.5g/day) vs. usual intake	−5.12 (−6.27 to −3.96)	Yes	He 2013
Hypertensives (Black)	Low sodium intake vs. high sodium intake	-6.64 (-9.00 to -4.27)	Yes	Graudal 2020
	Reduced sodium intake (-3.9g/day) vs. usual intake	−7.83 (−10.96 to −4.71)	Yes	He 2013
Hypertensives (Asian)	Low sodium intake vs. high sodium intake	-7.75 (-11.44, -4.07)	Yes	Graudal 2020
	Reduced sodium intake (-4.0g/day) vs. usual intake	−5.41 (−9.27 to −1.56)	Yes	He 2013
Non-hypertensives	Reduced sodium intake vs. usual intake	–1.38 (-2.74, -0.02)	No	WHO 2012
	Reduced sodium intake (-4.4g/day) vs. usual sodium intake	−2.42 (−3.56, −1.29)	Yes	He 2013
	Reduced sodium intake vs. usual intake	-0.66 (-0.93 to -0.39)	No	Huang 2020
Non-hypertensives (White)	Reduced sodium intake (-4.5g/day) vs. usual intake	−2.11 (−3.03 to −1.19)	Yes	He 2013
	Low sodium intake vs. high sodium intake	-1.14 (-1.65 to -0.63)	No	Graudal 2020
Non-hypertensives (Black)	Reduced sodium intake (-2.4g/day) vs. usual intake	−4.02 (−7.37 to −0.68)	Yes	He 2013

	Low sodium intake vs. high sodium intake	-5.71 (-6.67 to -4.74)	Yes	Graudal 2020
Non-hypertensives (Asian)	Low sodium intake vs. high sodium intake	-1.50(-3.09 to 0.10)	No	Graudal 2020

Table S4: The impact of sodium changes on diastolic blood pressure

Description of the population	Description of change in exposure	Change in Diastolic Blood Pressure (mmHg)	Clinically meaningful difference	Reference
General Population	Reduced sodium intake vs. usual sodium intake.	-1.54 (-2.11, -0.98)	No	WHO 2012
	Reduced sodium intake (-4.4g/day) vs. usual sodium intake	-2.06 (-2.67, -1.45)	Yes	He 2013
	Reduced sodium intake vs. usual sodium intake	-2.07 (-2.48, -1.67)	Yes	Huang 2020
Hypertensives	Reduced sodium intake vs. usual intake	-2.26 (-3.02, -1.50)	Yes	WHO 2012
	Reduced sodium intake (-4.4g/day) vs. usual intake	-2.82 (-3.54, -2.11)	Yes	He 2013
	Reduced sodium intake	-1.37 (-1.65, -1.09)	No	Huang 2020
	Low Sodium diet	-0.73 (-3.14, 1.68)	No	Schwingschakl 2019
Hypertensives (White)	Low sodium intake vs. high sodium intake	-2.87 (-3.41, -2.32)	Yes	Graudal 2020
	Reduced sodium intake)-4.5g/day) vs. usual intake	-2.66 (-3.37, -1.95)	Yes	He 2013
Hypertensives (Black)	Low sodium intake vs. high sodium intake	-2.91 (-4.52, -1.30)	Yes	Graudal 2020
	Reduced sodium intake (-3.9g/day) vs. usual intake	-4.08 (-5.90, -2.26)	Yes	He 2013
Hypertensives (Asian)	Low sodium intake vs. high sodium intake	-2.68 (-4.21, -1.15)	Yes	Graudal 2017
	Reduced sodium intake (-4.0g/day) vs. usual intake	-2.17 (-4.31, -0.03)	Yes	He 2013
Non-hypertensives	Reduced sodium intake vs. usual intake	-0.58 (-1.29, 0.01)	No	WHO 2012
	Reduced sodium intake (-4.4g/day) vs. usual sodium intake	-1.00 (-1.85, -0.15)	No	He 2013
	Reduced sodium intake vs. usual intake	-0.21 (-0.41,-0.02)	No	Huang 2020
Non-hypertensives (White)	Reduced sodium intake (-4.5g/day) vs. usual intake	-0.88 (-1.68, -0.08)	No	He 2013
	Low sodium intake vs. high sodium intake	0.01 (-0.37, 0.39)	No	Graudal 2020
Non-hypertensives (Black)	Reduced sodium intake (-2.4g/day) vs. usual intake	-1.98 (-4.45, 0.49)	No	He 2013

	Low sodium intake vs. high sodium intake	-2.01 (-4.37, 0.35)	No	Graudal 2020
Non-hypertensives (Asian)	Low sodium intake vs. high sodium intake	-1.06 (-2.53, 0.41)	No	Graudal 2020

Table S5: The impact of all fats on Systolic Blood Pressure

Population	Description of change in exposure	Change in Systolic blood pressure	Clinically meaningful difference	Reference
General population	Low (<30% TEI) vs. high fat (>30% TEI) diets	1.55 (-0.38, 3.47)	No	Lu 2018
	Low fat diet (<30% TEI) vs low carbohydrate diet (<45% TEI)	1.0 (-1.50, 3.50)	No	Hu 2012
	Reduced Fat Intake (<30% TEI) vs usual diet	-0.56 (-1.52, 0.04)	No	Hooper 2012
Hypertension	Low fat diet (<30% TEI) vs. low carbohydrate diet (<25% TEI) vs	2.20 (-0.17, 4.56)	No	Schwingshackl 2019

Table S6: The impact of all fats on Diastolic Blood Pressure

Population	Description of change in exposure	Change in Diastolic blood pressure mmHg	Clinically meaningful difference	Reference
General population	Low (<30% TEI) vs. high fat (>30% TEI) diets	2.18 (0.74, 3.62)	Yes	Lu 2018
	Low fat diet (<30% TEI) vs low carbohydrate diet (<45% TEI)	0.7 (-0.2, 1.7)	No	Hu 2012
	Reduced Fat Intake	-0.25 (-0.96, 0.26)	No	Hooper 2012
Hypertension	Low fat diet (<30% TEI)	1.36 (-0.30, 3.01)	No	Schwingshackl 2019

Table S7: The impact of fatty acids on systolic blood pressure

Population	Description of change in exposure	Change in systolic blood pressure	Clinically meaningful difference	Reference
General population	Increase monounsaturated fatty acids to replace carbohydrate	-0.08 (-1.01, 0.84)	No	Jovanovski 2019
Diabetes	Increase mono-unsaturated fatty acids to replace carbohydrate	-2.31 (-4.13, -0.49)	Yes	Quian 2016
General Population	Increase polyunsaturated (omega-3) fatty acids (Food only)	0.10 (-1.31, 1.50)	No	Miller 2014
	Higher vs. lower polyunsaturated (omega-3 and omega-6) fatty acids	-0.47 (-2.2, 1.26)	No	Abelhamid 2018
	Increase polyunsaturated fat (omega-6) fatty acids to replace other dietary energy	-1.1 (-4.05, 1.85)	No	Hooper 2018
Hypertensive	Increase polyunsaturated (omega-3) fatty acids (Food and supplement)	-4.51 (-6.12, -2.83)	Yes	Miller 2014
Normotensive	Increase polyunsaturated (omega-3) fatty acids (Food and supplement)	-1.25 (-2.05, -0.46)	No	Miller 2014
Diabetes	Increase polyunsaturated (omega-3) fatty acids	-2.1 (-4.48, 0.28)	No	Chewcharat 2020
	Increase polyunsaturated (omega-3) fatty acids	-0.00 (-0.15, 0.14)	No	O'Mahoney 2018
	Increase polyunsaturated (omega-3) fatty acids	-1.69 (-5.04, 1.65)	No	Hartweg 2007
General Population	Increase saturated fat to replace any foods	-0.19 (-1.36, 0.97)	No	Hooper 2020

Table S10: The impact of fatty acids on diastolic blood pressure

Population	Description of change in exposure	Change in diastolic blood pressure (mmHg)	Clinically meaningful difference	Reference
General population	Increase monounsaturated fatty acids to replace carbohydrate	0.01 (-0.73, 0.75)	No	Jovanovski 2019
Diabetes	Increase mono-unsaturated fatty acids to replace carbohydrate	-2.64 (-5.91, 0.63)	No	Quian 2016
General Population	Increase polyunsaturated (omega-3) fatty acids (Food only)	-0.38 (-1.46, 0.70)	No	Miller 2014
	Higher vs. lower polyunsaturated (omega-3 and omega-6) fatty acids	0.24 (-0.55, 1.02)	No	Abelhamid 2018
	Increase polyunsaturated fat (omega-6) fatty acids to replace other dietary energy	-0.30 (-2.04, 1.44)	No	Hooper 2018
Hypertensive	Increase polyunsaturated (omega-3) fatty acids (Food and supplement)	-3.05 (-4.35, -1.74)	Yes	Miller 2014
Normotensive	Increase polyunsaturated (omega-3) fatty acids (Food and supplement)	-0.62 (-1.22, -0.02)	No	Miller 2014
Diabetes	Increase polyunsaturated (omega-3) fatty acids	1.04 (1.81, 3.89)	No	Chewcharat 2020
	Increase polyunsaturated (omega-3) fatty acids	0.04 (-0.08, 0.17)	No	O'Mahoney 2018
	Increase polyunsaturated (omega-3) fatty acids	-1.79 (-3.56, -0.02)	No	Hartweg 2007
General Population	Increase saturated fat to replace any foods	-0.36 (-1.03, 0.32)	No	Hooper 2020

Table S8: The impact of low carbohydrate diets on Systolic Blood Pressure

Population	Description of change in exposure	Change in Systolic blood pressure	Clinically meaningful difference	Reference
General population	Low carbohydrate diet (10-45% TEI) vs. high carbohydrate diet (45-55% TEI)	-1.10 (-2.53, 0.33)	No	Fechner 2020
	Low carbohydrate diet vs. high carbohydrate diet	-1.41 (-2.26, -0.56)	No	Dong 2020
	Low carbohydrate diet vs high carbohydrate diet	-4.81 (-5.33, -4.29)	Yes	Santos 2012
	Low carbohydrate diet (<45% TEI) vs. low fat diet (<30% TEI)	-1.00 (-3.5, 1.5)	No	Hu 2012
Hypertension	Low carbohydrate diet (<25% TEI) vs low fat diet (<30% TEI)	-2.20 (-4.56, 0.17)	No	Schwingshackl 2019
Diabetes	Low carbohydrate diet (5-40% TEI) vs control (40-60% TEI)	-0.93 (-2.24, 0.37)	No	Korsmo-Haugen 2019
	Low carbohydrate diet vs usual diet (<50% TEI) vs other dieting approaches	-2.74 (-5.27, -0.20)	Yes	Huntris 2018
General population	Moderate-low carbohydrate diet (40-45% TEI) vs control (45-55% TEI)	-4.05 (-4.99, -3.10)	Yes	Fechner 2020
General population	Low carbohydrate diet (30-40% TEI) vs control (45-55% TEI)	-0.40 (-2.24, 1.44)	No	Fechner 2020
General population	Very low carbohydrate diet (<30% TEI) vs control (45-55% TEI)	-0.21 (-3.06, 2.63)	No	Fechner 2020
	Very-low-carbohydrate ketogenic diets (<50g carbohydrate) vs restricted energy diet (<30% TEI from fat)	-1.47 (-3.44, 0.50)	No	Bueno 2013

Table S9: The impact of low carbohydrate diet on Diastolic Blood Pressure

Population	Description of change in exposure	Change in Diastolic blood pressure	Clinically meaningful difference	Reference
General population	Low carbohydrate diet (10-45% TEI) vs. high carbohydrate diet (45-55% TEI)	-1.07 (-2.10, -0.05)	No	Fechner 2020
	Low carbohydrate diet (<40% TEI) vs. high carbohydrate diet (45-55% TEI)	-1.71 (-2.26, -0.56)	No	Dong 2020
	Low carbohydrate diet vs high carbohydrate diet	-3.10 (-3.45, -2.74)	Yes	Santos 2012
	Low carbohydrate diet (<45% TEI) vs. low fat diet (<30% TEI)	-0.7 (-1.6, 0.2)	No	Hu 2012
Hypertension	Low carbohydrate diet (<25% TEI) vs low fat diet (<30% TEI)	-1.36 (-3.01, 0.30)	No	Schwingshackl 2019
Diabetes	Low carbohydrate diet (5-40% TEI) vs control (40-60% TEI)	-0.21 (-1.20, 0.75)	No	Korsmo-Haugen 2019
	Low carbohydrate diet vs usual diet (<50% TEI) vs other dieting approaches	-0.99 (-2.24, 0.25)	No	Huntris 2018
General population	Moderate-low carbohydrate diet (40-45% TEI) vs control (45-55% TEI)	-2.64 (-3.32, -1.96)	Yes	Fechner 2020
General population	Low carbohydrate diet (30-40% TEI) vs control (45-55% TEI)	-1.31 (-2.79, 0.17)	No	Fechner 2020
General population	Very low carbohydrate diet (<30% TEI) vs control (45-55% TEI)	1.53 (-1.20, 4.25)	No	Fechner 2020
	Very-low-carbohydrate ketogenic diets (<50g carbohydrate) vs restricted energy diet (<30% TEI from fat)	-1.43 (-2.49, -0.37)	No	Bueno 2013

Table S11: The impact of fibre changes on systolic blood pressure

Population	Description of change in exposure	Change in systolic blood pressure	Clinically meaningful difference	Reference
General population	Higher vs lower dietary fibre	-1.27 (-2.50, -0.04)	No	Reynolds 2019
	Increase in soluble fibre intake vs. other foods	-1.59 (-2.72, -0.46)	No	Khan 2018
	Increase in dietary fibre intake vs. other foods	-1.57 (-4.45, 1.31)	No	Hartley 2016
Hypertensive	Increase in soluble fibre intake vs. other foods	-3.23 (-5.33, -1.14)	Yes	Khan 2018
Systolic Blood Pressure>130	Increase in soluble fibre intake vs. other foods	-2.68 (-4.69, -0.67)	Yes	Khan 2018
Healthy patients	Increase in soluble fibre intake vs. other foods	-2.77 (-5.65, 0.11)	Yes	Khan 2018
Systolic blood pressure <130	Increase in soluble fibre intake vs. other foods	-0.28 (-2.45, 1.89)	No	Khan 2018

Table S12: The impact of fibre changes on diastolic blood pressure

Population	Description of change in exposure	Change in diastolic blood pressure	Clinically meaningful difference	Reference
General population	Increase in soluble fibre intake (g) vs. other foods	-0.39 (-0.76, -0.01)	No	Khan 2018
	Increase in dietary fibre intake (g) vs. other foods	-2.40 (-3.57, -1.23)	Yes	Hartley 2016
	Higher vs lower dietary fibre	-1.34 (-2.96 ,0.27)	No	Reynolds 2019
Hypertensive	Increase in soluble fibre intake (g) vs. other foods	-1.22 (-2.38, -0.07)	No	Khan 2018
Diastolic Blood Pressure>85	Increase in soluble fibre intake (g) vs. other foods	-0.96 (-3.22, 1.30)	No	Khan 2018
Healthy patients	Increase in soluble fibre intake (g) vs. other foods	-0.34 (-1.65, 0.96)	No	Khan 2018
Diastolic Blood Pressure<85	Increase in soluble fibre intake (g) vs. other foods	0.19 (-1.26, 1.64)	No	Khan 2018

Table S13: The impact of free sugars on Systolic Blood Pressure

Population	Description of change in exposure	Change in Systolic Blood Pressure	Clinically meaningful difference	Reference
General population	Increased dietary free sugars (isocaloric)	-0.24 (-2.38, 1.90)	No	Te Morenga 2014
General population	Replace carbohydrate with fructose	-1.10 (-2.64, 0.44)	No	Ha 2012
Diabetes	Replace carbohydrate with fructose	2.03 (-11.55, 15.62)	No	Ha 2012

Table S14: The impact of free sugars on Diastolic Blood Pressure

Population	Description of change in exposure	Change in Diastolic Blood Pressure	Clinically meaningful difference	Reference
General population	Increased dietary free sugars (isocaloric)	0.65 (-0.31, 1.61)	No	Te Morenga 2014
General population	Replace carbohydrate with fructose	-1.54 (-2.77, -0.32)	No	Ha 2012
Diabetes	Replace carbohydrate with fructose	-2.00 (-7.54, 3.54)	No	Ha 2012

Table S15: The impact of sodium changes on total cholesterol

Description of the population	Description of change in exposure	Change in Total Cholesterol mmol/L	Clinically meaningful difference	Reference
General Population	Reduced sodium intake vs. usual intake	0.02 (-0.03, 0.07)	No	WHO 2012
	Reduced sodium intake vs. usual intake	0.05 (-0.02, 0.11)	No	He 2013
	Low sodium intake vs. high sodium intake	0.13 (0.05, 0.22)^	Yes	Graudal 2020
Hypertension	Low sodium intake vs. high sodium intake	0.15 (0.05, 0.26)^	Yes	Graudal 2020
	Reduced sodium intake vs. usual intake	0.01 (-0.16, 0.17)	No	WHO 2012
Non-hypertension	Reduced sodium intake vs. usual intake	0.10 (-0.03, 0.23)^	No	Graudal 2020

^ Results converted mmol/l=0.02586*mg/dL

Table S16: The impact of sodium changes on HDL cholesterol

Description of the population	Description of change in exposure	Change in HDL Cholesterol mmol/L	Clinically meaningful difference	Reference
General Population	Reduced sodium intake vs. usual intake	-0.01 (-0.03, 0.00)	No	WHO 2012
	Reduced sodium intake (-4.4g/day) vs. usual intake	-0.02 (-0.06, 0.01)	No	He 2013
	Low sodium intake vs. high sodium intake	-0.01 (-0.04, 0.03)^	No	Graudal 2020
Hypertension	Low sodium intake vs. high sodium intake	-0.06 (-0.12, 0.00)	No	WHO 2012
	Low sodium intake vs. high sodium intake	-0.03 (-0.09, 0.04)^	No	Graudal 2020
Non-hypertension	Reduced sodium intake vs. usual intake	-0.01 (-0.03, 0.00)	No	WHO 2012
	Low sodium intake vs. high sodium intake	0.00 (-0.04, 0.04)^	No	Graudal 2020
^ Results converted mmol/l=0.02586*mg/dL				

Table S17: The impact of sodium changes on LDL cholesterol

Description of the population	Description of change in exposure	Change in HDL Cholesterol mmol/L	Clinically meaningful difference	Reference
General Population	Reduced sodium intake vs. usual intake	0.03 (-0.02, 0.08)	No	WHO 2012
	Reduced sodium intake (-4.4g/day) vs. usual intake	0.05 (-0.01, 0.12)	No	He 2013
	Low sodium intake vs. high sodium intake	0.06 (-0.03, 0.15)^	No	Graudal 2020
Hypertension	Reduced sodium intake vs. usual intake	0.08 (-0.11, 0.27)	No	WHO 2012
	Low sodium intake vs. high sodium intake	0.07 (-0.04, 0.17)^	No	Graudal 2020
Non-hypertension	Reduced sodium intake vs. usual intake	0.13 (-0.27, 0.54)	No	WHO 2012
	Low sodium intake vs. high sodium intake	0.06 (-0.10, 0.22)^	No	Graudal 2020
^ Results converted mmol/l=0.02586*mg/dL				

Table S18: The impact of all fats on Total Cholesterol

Population	Description of change in exposure	Change in Total Cholesterol mmol/l	Clinically meaningful difference	Reference
General population	Reduced Fat Intake (<30% TEI) and replace with carbohydrate	-0.1 (-0.14, -0.05)	Yes	Hooper 2012
	Low fat diet (<30% TEI) vs low carbohydrate diet (<45% TEI)	-0.07 (-0.12, -0.02)^	No	Hu 2012
	Low vs. high fat diets (no energy restriction)	-0.08 (-0.23, 0.07)^	No	Lu 2018
	Low fat diet (<35% TEI from fat >50% Carbohydrate) vs carbohydrate restricted diet	-0.01 (-0.04, 0.03)	No	Gjuladin-Hellon 2019
Diabetes	Low fat (<30% TEI) vs. regular diet	-0.24 (-0.99, 0.50)	No	Pan 2019
	Low fat (<30% TEI) vs. low carbohydrate (<45% TEI)	0.09 (-0.16, 0.33)	No	Pan 2019

^ Results converted mmol/l=0.02586*mg/dL

Table S19: The impact of total fats on HDL Cholesterol

Population	Description of change in exposure	Change in HDL Cholesterol mmol/l	Clinically meaningful difference	Reference
General population	Reduced Fat Intake (<30% TEI) and replace with carbohydrate	-0.01 (-0.02, 0.01)	No	Hooper 2012
	Low fat diet (<30% TEI) vs low carbohydrate diet (<45% TEI)	-0.09 (-0.12, -0.05)^	No	Hu 2012
	Low vs. high fat diets (no energy restriction)	-0.03 (-0.09, -0.03)	No	Lu 2018
	Low fat diet (<35% TEI from fat >50% Carbohydrate) vs carbohydrate restricted diet	-0.08 (-0.11, -0.06)	No	Gjuladin-Hellon 2019
Diabetes	Low fat (<30% TEI) vs. low carbohydrate (<45% TEI)	0.10 (0.03, 0.17)	Yes	Pan 2019
	Low fat (<30% TEI) vs. regular diet	-0.04 (-0.22, 0.15)	No	Pan 2019
	Low fat diet (<30% TEI) vs control	-0.03 (-0.09, 0.05)	No	Neuenschwander 2019
^ Results converted mmol/l=0.02586*mg/dL				

Table S20: The impact of all fats on LDL Cholesterol

Population	Description of change in exposure	Change in LDL Cholesterol mmol/l	Clinically meaningful difference	Reference
General population	Reduced Fat Intake (<30% TEI) and replace with carbohydrate	-0.10 (-0.14, -0.05)	Yes	Hooper 2012
	Low fat diet (<30% TEI) vs low carbohydrate diet (<45% TEI)	-0.10 (-0.17, -0.03)^	Yes	Hu 2012
	Low vs. high fat diets (no energy restriction)	-0.01 (-0.36, 0.34)	No	Lu 2018
	Low fat diet (<35% TEI from fat >50% Carbohydrate) vs carbohydrate restricted diet	-0.07 (-0.13, 0.02)	No	Gjuladin-Hellon 2019
Diabetes	Low fat (<30% TEI) vs. low carbohydrate (<45% TEI)	-0.07 (-0.32, 0.18)	No	Pan 2019
	Low fat (<30% TEI) vs. regular diet	-0.10 (-0.80, 0.60)	No	Pan 2019
	Low fat diet (<30% TEI) vs control	-0.11 (-0.24, 0.02)	No	Neuenschwander 2019

^ Results converted mmol/l=0.02586*mg/dL

Table S21: The impact of fatty acids on Total Cholesterol

Population	Description of change in exposure	Change in Total Cholesterol	Clinically meaningful difference	Reference
General population	Increase monounsaturated fat to replace saturated fat	-0.05 (-0.05, -0.04)	No	Mensink 2016
General population	Increase in polyunsaturated (omega-3 or omega-6) fatty acids to replace saturated fat	-0.32 (-0.50, -0.14)	Yes	Abelhamid 2018
	Increase polyunsaturated fat (omega-3 or omega-6) to replace saturated fat	-0.06 (-0.07, -0.06)	No	Mensink 2016
	Increase polyunsaturated fat to replace saturated fat	-0.33 (-0.47, -0.19)	Yes	Hooper 2020
General population	Increase in polyunsaturated (omega-3 or omega-6) fatty acids to replace monounsaturated fat	-0.17 (-0.33, -0.00)	Yes	Abelhamid 2018
General population	Increase in polyunsaturated (omega-3 or omega-6) fatty acids to replace other dietary energy	-0.12 (-0.23, -0.02)	Yes	Abelhamid 2018
	Increase polyunsaturated fat (omega-6) fatty acids to replace other dietary energy	-0.33 (-0.5, -0.16)	Yes	Hooper 2018
Diabetes	Increase polyunsaturated fat (omega-3) fatty acids	0.10 (-0.12, 0.31)^	No	Chewcharat 2020
	Increase polyunsaturated fat (omega-3) fatty acids	-0.23 (-1.14, 0.68)	No	Natto 2019
General Population	Increase saturated fat to replace carbohydrate	0.05 (0.04, 0.05)	No	Mensink 2016
	Increase saturated fat to replace carbohydrate	0.18 (0.04, 0.32)	Yes	Hooper 2020
	Increase saturated fat to replace any foods	0.24 (0.12, 0.36)	Yes	Hooper 2020

^ Results converted mmol/l=0.02586*mg/dL

Table S22: The impact of fatty acids on HDL Cholesterol

Population	Description of change in exposure	Change in HDL Cholesterol	Clinically meaningful difference	Reference
General population	Increase monounsaturated fat to replace saturated fat	-0.002 (-0.004, -0.000)	No	Mensink 2016
Diabetes	Increase in monounsaturated fatty acids to replace carbohydrate	0.06 (0.02, 0.10)	No	Quian 2016
General population	Increase in total polyunsaturated (omega-3 or omega-6) fatty acids	0.00 (-0.02,0.02)	No	Brown 2019
	Increase in polyunsaturated (omega-3 or omega-6) fatty acids to replace saturated fat	-0.01 (-0.03, 0.02)	No	Abelhamid 2018
	Increase polyunsaturated fat (omega-3 or omega-6) to replace saturated fat	-0.005 (-0.006, -0.003)	No	Mensink 2016
	Increase polyunsaturated fat to replace saturated fat	-0.01 (-0.04, 0.01)	No	Hooper 2020
General population	Increase in polyunsaturated (omega-3 or omega-6) fatty acids to replace monounsaturated fat	-0.01 (-0.03, 0.02)	No	Abelhamid 2018
Diabetes	Increase in polyunsaturated fatty acids to replace monounsaturated fat	-0.04 (-0.15, 0.07)	No	Quian 2016
General population	Increase in polyunsaturated (omega-3 or omega-6) fatty acids to replace other dietary energy	-0.01 (-0.02, 0.01)	No	Abelhamid 2018
	Increase in polyunsaturated (omega-6) fatty acids to replace other dietary energy	-0.01 (-0.03, 0.02)	No	Hooper 2018
Diabetes	Increase in polyunsaturated fatty acids (omega-3) fatty acids to replace other dietary energy	0.12 (0.02, 0.22)^	Yes	Chewcharat 2020
	Increase in polyunsaturated fatty acids (omega-3) fatty acids to replace other dietary energy	-0.06 (-0.10, -0.02)	No	Natto 2019
General population	Increase saturated fat to replace carbohydrate	0.01 (0.01, 0.01)	No	Mensink 2016
	Increase saturated fat to replace carbohydrate	0.01 (0.00, 0.03)	No	Hooper 2020
	Increase saturated fat to replace other diet	0.01 (-0.01, 0.02)	No	Hooper 2020
^ Results converted mmol/l=0.02586*mg/dL				

Table S23: The impact of fatty acids on LDL Cholesterol

Population	Description of change in exposure	Change in LDL Cholesterol	Clinically meaningful difference	Reference
General population	Increase monounsaturated fatty acids to replace saturated fat	-0.04 (-0.05, -0.04)	No	Mensink 2016
Diabetes	Increase in monounsaturated fatty acids to replace carbohydrate	0.05 (-0.07, 0.16)	No	Quian 2016
General population	Increase in total polyunsaturated (omega-3 or omega-6) fatty acids	-0.12 (-0.41, 0.17)	No	Brown 2019
	Increase in polyunsaturated (omega-3 or omega-6) fatty acids to replace saturated fat	-0.04 (-0.21, 0.14)	No	Abelhamid 2018
	Increase polyunsaturated fat (omega-3 or omega-6) to replace saturated fat	-0.06 (-0.06, -0.05)	No	Mensink 2016
	Increase polyunsaturated fat to replace saturated fat	-0.48 (-0.90, -0.06)	No	Hooper 2020
General population	Increase in polyunsaturated (omega-3 or omega-6) fatty acids to replace monounsaturated fat	-0.00 (-0.12, 0.12)	No	Abelhamid 2018
Diabetes	Increase in polyunsaturated fatty acids to replace monounsaturated fat	0.15 (-0.14, 0.44)	No	Quian 2016
General population	Increase in polyunsaturated (omega-3 or omega-6) fatty acids to replace other dietary energy	-0.01 (-0.09, 0.06)	No	Abelhamid 2018
	Increase polyunsaturated fat (omega-6) fatty acids to replace other dietary energy	-0.04 (-0.21, 0.14)	No	Hooper 2018
Diabetes	Increase in polyunsaturated fatty acids (omega-3) fatty acids	0.06 (-0.06, 0.18)^	No	Chewcharat 2020
	Increase in polyunsaturated fatty acids (omega-3) fatty acids*	-0.10 (-0.17, -0.03)	Yes	O'Mahoney 2018
	Increase in polyunsaturated fatty acids (omega-3) fatty acids*	-0.19 (-0.50, 0.12)	No	Natto 2019
General population	Increase saturated fat to replace carbohydrate	0.03 (0.03, 0.04)	No	Mensink 2016
	Increase saturated fat to replace carbohydrate	0.16 (-0.04, 0.36)	No	Hooper 2020
General population	Increase saturated fat to replace other diets	0.19 (0.05, 0.33)	No	Hooper 2020

* Review included studies of diet and capsule interventions
^ Results converted mmol/l=0.02586*mg/dL

Table S24: The impact of low carbohydrate diets on Total Cholesterol

Population	Description of change in exposure	Change in Total Cholesterol	Clinically meaningful difference	Reference
General population	Low carbohydrate diet vs. high carbohydrate diet	0.13 (0.08, 0.19)	Yes	Dong 2020
	Low carbohydrate diet (<45% TEI) vs. high carbohydrate diet (45-55% TEI)	0.10 (0.01, 0.18)	Yes	Fechner 2020
	Low carbohydrate diet (<45% TEI) vs. low fat diet (<30% TEI)	0.07 (0.02, 0.12)^	No	Hu 2012
Diabetes	Low carbohydrate diet (5-40% TEI) vs control (40-60% TEI)	0.04 (-0.12, 0.2)	No	Korsmo-Haugen 2019
	Low carbohydrate diet vs usual diet	-0.08 (-0.23, 0.08)	No	Huntris 2018
General population	Moderate-low carbohydrate diet (40-45% TEI) vs control (45-55% TEI)	-0.05 (-0.19, 0.08)	No	Fechner 2020
General population	Low carbohydrate diet (30-40% TEI) vs control (45-55% TEI)	0.21 (0.03, 0.39)	Yes	Fechner 2020
Diabetes	Low carbohydrate diet vs low fat diet	-0.09 (-0.33, 0.16)	No	Pan 2019
General population	Very low carbohydrate diet (<30% TEI) vs control (45-55% TEI)	0.26 (-0.02, 0.54)	No	Fechner 2020
Diabetes	Very low carbohydrate (<26% TEI) vs regular diet	-0.24 (-0.99, 0.50)	No	Pan 2019

Table S25: The impact of low carbohydrate diets on HDL Cholesterol

Population	Description of change in exposure	Change in HDL Cholesterol	Clinically meaningful difference	Reference
General population	Low carbohydrate diet vs. high carbohydrate diet	0.10 (0.08, 0.12)	No	Dong 2020
	Low carbohydrate diet vs high carbohydrate diet	0.04 (0.03, 0.05)^	No	Santos 2012
	Low carbohydrate diet (<45% TEI) vs. low fat diet (<30% TEI) vs	0.09 (0.05, 0.12)^	No	Hu 2012
General population	Low carbohydrate diet (<45% TEI) vs control (45-55% TEI)	0.08 (0.04, 0.13)	No	Fechner 2020
Diabetes	Low carbohydrate diet (5-40% TEI) vs control (40-60% TEI)	0.04 (-0.01, 0.1)	No	Korsmo-Haugen 2019
	Low carbohydrate diet (<50% TEI) vs usual diet	0.06 (0.04, 0.09)	No	Huntris 2018
General population	Moderate-low carbohydrate diet (40-45% TEI) vs control (45-55% TEI)	0.05 (-0.05, 0.15)	No	Fechner 2020
General population	Low carbohydrate diet (30-40% TEI) vs control (45-55% TEI)	0.08 (0.05, 0.11)	No	Fechner 2020
General population	Very low carbohydrate diet (<30% TEI) vs control (45-55% TEI)	0.13 (0.11, 0.16)	Yes	Fechner 2020
	Very-low-carbohydrate ketogenic diets (<50g carbohydrate) vs restricted energy diet (<30% TEI from fat)	0.09 (0.06, 0.12)	No	Bueno 2013
Diabetes	Very low carbohydrate (<26% TEI) vs regular diet	-0.04 (-0.22, 0.15)	No	Pan 2019
	Very low carbohydrate diet (<25% TEI) vs usual diet	0.06 (-0.1, 0.12)	No	Neuenschwander 2019
Diabetes	Low carbohydrate diet (<26% TEI) vs low fat diet	-0.10 (-0.17, -0.03)	Yes	Pan 2019
^ Results converted mmol/l=0.02586*mg/dL				

Table S26: The impact of low carbohydrate diet on LDL Cholesterol

Population	Description of change in exposure	Change in LDL Cholesterol	Clinically meaningful difference	Reference
General population	Low carbohydrate diet (10-45% TEI) vs. high carbohydrate diet (45-55% TEI)	0.10 (0.02, 0.17)	Yes	Fechner 2020
	Low carbohydrate diet vs. high carbohydrate diet	0.11 (0.02, 0.19)	Yes	Dong 2020
	Low carbohydrate diet vs high carbohydrate diet	-0.07 (-0.13, -0.01)	No	Santos 2012
	Low carbohydrate diet (<45% TEI) vs. low fat diet (<30% TEI) vs	0.10 (0.03, 0.17)^	Yes	Hu 2012
Diabetes	Low carbohydrate diet (5-40% TEI) vs control (40-60% TEI)	-0.01 (-0.13, 0.11)	No	Korsmo-Haugen 2019
	Low carbohydrate diet (<50% TEI) vs usual diet	0.05 (-0.1, 0.19)	No	Huntris 2018
General population	Moderate-low carbohydrate diet (40-45% TEI) vs control (45-55% TEI)	0.01 (-0.16, 0.17)	No	Fechner 2020
General population	Low carbohydrate diet (30-40% TEI) vs control (45-55% TEI)	0.11 (0.02, 0.20)	Yes	Fechner 2020
General population	Very low carbohydrate diet (<30% TEI) vs control (45-55% TEI)	0.32 (0.08, 0.56)	Yes	Fechner 2020
	Very-low-carbohydrate ketogenic diets (<50g carbohydrate) vs restricted energy diet (<30% TEI from fat)	0.12 (0.04, 0.20)	Yes	Bueno 2013
Diabetes	Low carbohydrate diet (<25% TEI) vs usual diet	0.12 (0.04, 0.02	Yes	Neuenschwander 2019

Table S27: The impact of fibre changes on Total Cholesterol

Population	Description of change in exposure	Change in Total Cholesterol	Clinically meaningful difference	Reference
General population	Increase in dietary fibre intake (15g/day) vs. other foods	-0.16 (-0.42, 0.09)	No	Hartley 2016
	Higher vs lower dietary fibre	-0.15 (-0.22, -0.07)	Yes	Reynolds 2019
Diabetes	Increase in soluble fibre intake (Psyllium 9g/day) vs. other foods	-0.21 (-0.50, 0.09)^	No	Xiao 2020
^ Results converted mmol/l=0.02586*mg/dL				

Table S28: The impact of fibre changes on HDL Cholesterol

Population	Description of change in exposure	Change in HDL Cholesterol	Clinically meaningful difference	Reference
General population	Increase in soluble fibre intake (15g day) vs. other foods	-0.03 (-0.07, 0.02)	No	Hartley 2016
	Higher vs lower dietary fibre	0.01 (-0.01, 0.03)	No	Reynolds 2019
Diabetes	Increase in fibre intake (Psyllium 9g/day) vs. other foods	0.12 (-0.06, 0.31)^	No	Xiao 2020
* Results converted mmol/l=0.02586*mg/dL				

Table S29: The impact of fibre changes on LDL Cholesterol

Population	Description of change in exposure	Change in HDL Cholesterol	Clinically meaningful difference	Reference
General population	Increase in dietary fibre intake (15g/day) vs. other foods	-0.14 (-0.26, -0.03)	Yes	Hartley 2016
	Higher vs lower dietary fibre	-0.10 (-0.15, -0.04)	Yes	Reynolds 2019
Diabetes	Increase in fibre intake (Psyllium 9g/day) vs. other foods	-0.23 (-0.35, -0.12)	Yes	Xiao 2020
* Results converted mmol/l=0.02586*mg/dL				

Table S30: The impact of free sugars on Total Cholesterol

Population	Description of change in exposure	Change in total Cholesterol	Clinically meaningful difference	Reference
General population	Increased dietary free sugars (isocaloric)	0.23 (0.12, 0.34)	Yes	Te Morenga 2014
Diabetes	Replace starch with fructose	-0.02 (-0.18, 0.14)	No	Sievenpiper 2009

Table S31: The impact of free sugars on HDL Cholesterol

Population	Description of change in exposure	Change in HDL Cholesterol	Clinically meaningful difference	Reference
General population	Increased dietary free sugars (isocaloric)	0.02 (0.00, 0.04)	No	Te Morenga 2014
Diabetes	Replace starch with fructose	0.02 (-0.05, 0.10)	No	Sievenpiper 2009

Table S32: The impact of free sugars and starch on LDL Cholesterol

Population	Description of change in exposure	Change in LDL Cholesterol	Clinically meaningful difference	Reference
General population	Replace sucrose with glucose (free sugars)	-0.06 (-0.20, 0.08)	No	Schwingshackl 2020
	Replace sucrose with fructose (free sugars)	-0.01 (-0.10, 0.09)	No	Schwingshackl 2020
	Replace glucose with starch (free sugars)	-0.17 (-0.36, 0.03)	No	Schwingshackl 2020
	Replace glucose with fructose (free sugars)	0.05 (-0.06, 0.16)	No	Schwingshackl 2020
	Replace starch with sucrose (free sugars)	0.23 (0.07, 0.38)	Yes	Schwingshackl 2020
	Replace starch with fructose (free sugars)	0.22 (0.05, 0.39)	Yes	Schwingshackl 2020
	Increased dietary free sugars (isocaloric)	0.17 (0.06, 0.28)	Yes	Te Morenga 2014
Diabetes	Replace starch with fructose	0.02 (-0.07, 0.11)	No	Sievenpiper 2009

Table S33: The impact of all fat changes on HbA1c

Population	Description of change in exposure	Change in HbA1c (%)	Clinically meaningful difference	Reference
Diabetes	Low fat (<30% TEI) vs. regular diet	-0.17 (-0.45, 0.21)	No	Pan 2019
	Low fat (<30% TEI) vs. control	-0.47 (-0.66, -0.28)	No	Schwingshackl 2018
	Low fat (<30% TEI) vs. low carbohydrate (<45% TEI)	-0.30 (-0.78, 1.38)	No	Pan 2019
	Low fat (<30% TEI) vs. low carbohydrate (<45% TEI)	0.35 (0.14, 0.56)	No	Schwingshackl 2018

Table S34: The impact of fatty acids on HbA1c

Population	Description of change in exposure	Change in HbA1c (%)	Clinically meaningful difference	Reference
General population	Increase monounsaturated fatty acids to replace carbohydrate	-0.09 (-0.12, -0.05)	No	Immamura 2016
	Increase monounsaturated fatty acids to replace saturated fatty fats	-0.12 (-0.19, -0.05)	No	Immamura 2016
Diabetes	Increase monounsaturated fatty acids to replace carbohydrate	-0.11 (-0.24, 0.02)	No	Quian 2016
General population	Increase polyunsaturated fatty acids to replace carbohydrate	-0.11 (-0.17, -0.05)	No	Immamura 2016
	Increase polyunsaturated fatty acids to replace saturated fatty fats	-0.15 (-0.23, -0.06)	No	Immamura 2016
	Increase polyunsaturated (omega-3) fatty acids	-0.02 (-0.07, 0.04)	No	Brown 2019
Diabetes	Increase polyunsaturated (omega-3) fatty acids	-0.03 (-0.45, 0.39)	No	Chewcharat 2020
	Increase polyunsaturated (omega-3) fatty acids*	-0.27 (-0.48, 0.06)	No	O’Mahoney 2018
	Increase polyunsaturated (omega-3) fatty acids*	-0.33 (-1.16, 0.5)	No	Natto 2019
General population	Increase polyunsaturated fatty acids to replace monounsaturated fatty acids	-0.03 (-0.09, 0.03)	No	Immamura 2016
General population	Increase saturated fatty acids to replace carbohydrate	0.03 (-0.02, 0.09)	No	Immamura 2016
* Review included studies of diet and capsule interventions				

Table S35: The impact of carbohydrates on HbA1c

Population	Description of change in exposure	Change in HbA1c	Clinically meaningful difference	Reference
General population	Low carbohydrate diet vs high carbohydrate diet	-0.21 (-0.24, -0.18)	No	Santos 2012
Diabetes	Low carbohydrate diet (5-40% TEI) vs control (40-60% TEI)	-0.09 (-0.11, 0.13)	No	Korsmo-Haugen 2019
	Low carbohydrate diet (6-45% TEI) vs high carbohydrate diet (>45% TEI)	-0.09 (-0.17, 0.08)	No	McArdle 2019
	Low carbohydrate diet (<50% TEI) vs other dieting approaches	-0.28 (-0.53, -0.02)	No	Huntriss 2018
	Low carbohydrate diet vs control	-0.82 (-1.11, -0.53)	Yes	Schwingshackl 2018
General population	Very-low-carbohydrate ketogenic diets (<50g carbohydrate) vs restricted energy diet (<30% TEI from fat)	-0.24 (-0.55, 0.06)	No	Bueno 2013
Diabetes	Very low carbohydrate diet (<26% TEI) vs regular diet	0.13 (-0.99, 1.25)	No	Pan 2019
	Very low carbohydrate diet (<26% TEI) vs high carbohydrate diet (>45% TEI)	-0.49 (-0.75, -0.23)	No	McArdle 2019
	Very Low carbohydrate diet (6-10% TEI) vs high carbohydrate diet (>45% TEI)	-0.13 (-0.34, 0.08)	No	McArdle 2019
Diabetes	Low carbohydrate diet (<26% TEI) vs high fat diet	0.30 (-0.78, 1.38)	No	Pan 2019

Table S36: The impact of fibre changes on HbA1c

Population	Description of change in exposure	Change in Total Cholesterol	Clinically meaningful difference	Reference
General Population	Higher vs lower dietary fibre	-0.35 (-0.73, 0.03)	No	Reynolds 2019
Diabetes	Increase in viscous fibre intake (10.9g/day) vs. control diet	-0.61 (-0.92, -0.29)	Yes	Jovanovski 2019
	Increase in fibre intake (Psyllium 9g/day) vs. control diet	-0.91 (-1.31, -0.51)	Yes	Xiao 2020

Table S36: The impact of free sugars on HbA1c

Population	Description of change in exposure	Change in HbA1c	Clinically meaningful difference	Reference
	Replace starch with sucrose	-0.30 (-1.99, 1.40)	No	Schwingshackl 2020
	Replace starch with glucose	0.08 (-1.21, 1.38)	No	Schwingshackl 2020
	Replace starch with fructose	-0.29 (-1.17, 0.58)	No	Schwingshackl 2020
	Increase small doses of fructose (median dose 36g/day)	-0.38 (-0.64,-0.13)	Yes	Norohna 2018
	Increase small doses of tagatose (median dose 45g/day)	-0.20 (-0.34, -0.06)	Yes	Norohna 2018
	Increase small doses of allulose (median dose 14g/day)	0.02 (-0.03, 0.07)	No	Norohna 2018
Diabetes	Replace carbohydrate consumption with fructose (median 60g/day)	-0.27 (-0.49, -0.04)	Yes	Cozma 2012

Methods for Evidence Synthesis and Causal Loop Model

Effects sizes were grouped and tabulated according to the nutrient groups and target population types used in the sampling framework matrix. On data extraction effect sizes were grouped according to other sub-population groupings (ethnicity), and nutrient exposures to reduce heterogeneity between studies. For example, differences in nutrient exposures (MUFA vs PUFA) or doses (low carbohydrate vs. very low carbohydrate) were separated.

As part of the narrative synthesis we categorised all associations between nutrients and health measures by the certainty of evidence and clinically meaningfully differences. High certainty evidence was determined if at least one study was graded as high quality using the AMSTAR2 checklist and there was no disagreement in the findings among studies of similar exposure and population. Moderate certainty evidence was determined if at least one study was graded moderate using the AMSTAR2 and there were no disagreements in evidence among studies of similar exposure and population. Low certainty evidence if all studies were graded low or critically low and/or there was disagreement among studies of similar exposure and population. Clinically significant improvement was defined for each risk factor based on the minimum change deemed clinically important; >2mm/Hg was used for systolic and diastolic blood pressure (16); 0.1mmol/L for total, LDL and HDL cholesterol; 0.5% change in HbA1c (17). Evidence was considered clinically significant if the mean exceeded these thresholds and the difference was statistically significant.

Our goal was to develop a conceptual model to synthesize existing knowledge surrounding the health outcomes of dietary changes and provide a causal structure for future modelling work. Primary nutrient groups were identified in the sampling framework matrix and the structure was developed incrementally by adding each additional nutrient group to the diagram. The magnitude of effects across

exposures were illustrated by the thickness of the line, and non-statistically significant results were indicated by a dashed line. In cases where evidence was conflicting, in terms of the direction of effect or statistical significance of the effect, the synthesis relied on a process of deliberation with consideration for the following criteria.

1. Review Quality (AMSTAR2)
2. Publication year
3. Study characteristics (reduce heterogeneity)
4. Number of studies in meta-analysis

All deliberations have been documented in the supplementary appendix with a rationale provided for the overall finding.

Table S38: Documented deliberations on conflicting evidence across studies for the casual loop diagram

Exposure	Outcome	Population	Disagreement	Decision	Reason
Sodium	Systolic Blood Pressure	Asian + Non-hypertensive	Statistical significance	Effect size is non-statistically significant	Graudal 2020 is a more recent source of evidence and the quality assessment found this to be a high quality study.
Sodium	Diastolic Blood Pressure	Non-hypertensive	Statistical significance and differences across ethnicity	Use pooled estimates for normotensive populations and assume statistical significance.	Outcomes consistently show negative effects for non-hypertensive. Non-statistical significance more commonly reported for ethnic subgroups, suggesting that sample sizes may not be large enough to reduce uncertainty. Prioritise evidence from normotensive populations.
Sodium	Total cholesterol	General Population	Statistical significance	Effect size is statistically significant	Graudal 2020 is a more recent source of evidence and the quality assessment found this to be a high quality study.
Sodium	Total cholesterol	Hypertensives	Statistical significance	Effect size is statistically significant	Graudal 2020 is a more recent source of evidence and the quality assessment found this to be a high quality study.

All fat	Systolic Blood Pressure	General Population	Direction of effect	Low fat diets increase systolic blood pressure and the effect is not statistically significant.	Differences are likely to be due to the differences in the comparator. Trials with a focus on substitution for carbohydrate are less likely to be confounded by the effects of weight loss. Prioritise evidence from substitution for carbohydrates.
All fat	Diastolic Blood Pressure	General Population	Direction and significance of effect	Low fat diets increase diastolic blood pressure and the effect is not statistically significant.	Differences are likely to be due to the differences in the comparator. Substitution for carbohydrate is preferred because the effects of a comparison with a usual diet may be mediated by weight loss. Prioritise evidence from substitution for carbohydrates.
All fat	Total Cholesterol	General population	Statistical significance	Low fat diets decrease total cholesterol and the effect is non-statistically significant	The effect size is not statistically significant in more recent studies with no energy restriction or substitution to carbohydrate. Prioritise publication year and substitution effects.
All fat	Total Cholesterol	Diabetes	Direction of effect	No difference in effects of total fat on total cholesterol in diabetes are observed.	The confidence intervals are large and overlap with the studies of the general population, and the study included fewer studies.
All fat	HDL Cholesterol	General population	Statistical significance	Low fat diets decrease HDL cholesterol and the effect is statistically significant	Only one study identified a non-significant association. Prioritise more recent evidence.
All fat	HDL Cholesterol	Diabetes	Direction of effect	No difference in effects of total fat on HDL cholesterol in diabetes are observed.	The confidence intervals are large and overlap with the studies of the general population, and the study included fewer studies.
All fat	LDL Cholesterol	General population	Statistical significance	Low fat diets decrease LDL cholesterol and the effect is non-statistically significant	The effect size is not statistically significant in more recent studies with no energy restriction or substitution to carbohydrate. Prioritise publication year and substitution effects.
All fat	HbA1c	Diabetes	Direction of effect and statistical significance	Total fat does not impact HbA1c	Differences are likely to be due to the differences in the comparator. Substitution for carbohydrate is preferred because the effects of a comparison with a usual diet may be mediated by weight loss. Prioritise evidence from substitution for carbohydrates in Schwingshakl (2020).
Fatty acids	Systolic Blood Pressure	General Population	Direction of effect and statistical significance	Polyunsaturated fatty acids reduces systolic blood pressure and the result is not statistically significant.	The studies each investigate the impact of omega-3, omega-6 and a combined analysis. This may explain the differences in

					findings. Prioritise review of both polyunsaturated fat types that combined evidence.
Fatty acids	Diastolic Blood Pressure	General Population	Direction of effect and statistical significance	Polyunsaturated fatty acids does not impact diastolic blood pressure	The studies each investigate the impact of omega-3, omega-6 and a combined analysis. This may explain the differences in findings. Prioritise review of both polyunsaturated fat types that combined evidence.
Fatty acids	Diastolic Blood Pressure	Diabetes	Direction of effect and statistical significance	Polyunsaturated fatty acids does not impact diastolic blood pressure	Chewcharat (2020) provide more recent evidence. However, large differences between studies suggests uncertainty.
Fatty acids	HDL cholesterol	Diabetes	Direction of effect and statistical significance	Polyunsaturated fat increases HDL cholesterol and the effect in non-statistically significant	Natto (2019) identified fewer and different studies in the review compared with Chewcharat (2020) and did not restrict studies to diet-only interventions. Prioritise large samples and diet only studies, but assume not significant to reflect inconsistencies across studies.
Fatty acids	LDL cholesterol	General population	Statistical significance	Polyunsaturated fat decreases LDL cholesterol, and the effect is statistically significant if replacing saturated fat.	Hooper (2020) and Mensink (2016) isolate the relationship by replacing saturated fat with carbohydrate and both identify a statistically significant association.
Fatty acids	LDL cholesterol	Diabetes	Direction of effect and statistical significance	No difference in effects of polyunsaturated fats on LDL cholesterol in diabetes	Conflicting and non-significant results suggest that there is weak evidence for an effect of polyunsaturated fat on LDL cholesterol in diabetes patients.
Fatty acids	LDL cholesterol	General population	Statistical significance	Saturated fat decreases LDL cholesterol and the relationship is statistically significant.	A stronger impact is observed when comparing with “other diets” may be due to the associated benefits of switches to polyunsaturated fat.
Fatty acids	HbA1c	General population	Statistical significance	Polyunsaturated fatty acids decrease HbA1c but the effect is non-statistically significant	Similar quality and number of studies included in the review. Prioritise Brown (2019) due to a more recent search date.
All carbohydrate	Systolic blood pressure	General population	Statistical significance	Carbohydrates decrease systolic blood pressure but the effect is non-statistically significant.	Prioritise the recent study with more studies added to the meta-analysis. No evidence of dose effect in Fetchner 2020 adds uncertainty in this effect.
All carbohydrate	Diastolic blood pressure	Diabetes	Statistical significance	Carbohydrates decrease systolic blood pressure in diabetes but the effect is non-statistically significant.	Prioritise the recent study with more studies added to the meta-analysis.

All carbohydrate	Diastolic blood pressure	General population	Statistical significance	Carbohydrates decrease diastolic blood pressure but the effect is non-statistically significant.	Differences between studies and wide confidence intervals indicates weaknesses in the data for the association between very low carbohydrate diets and diastolic blood pressure.
All carbohydrate	Total cholesterol	Diabetes	Direction of effect and statistical significance	No difference in effects of carbohydrates on total cholesterol in diabetes are observed.	Differences between studies and wide confidence intervals indicates a weakness in the data for the association between carbohydrates and cholesterol in diabetes.
All carbohydrate	HDL cholesterol	Diabetes	Direction of effect and statistical significance	No difference in effects of carbohydrates on HDL cholesterol in diabetes are observed.	Differences between studies and wide confidence intervals indicates a weakness in the data for the association between carbohydrates and cholesterol in diabetes.
All carbohydrate	LDL cholesterol	General population	Direction of effect	Carbohydrates decrease LDL cholesterol and the result is statistically significant.	Differences in effect size for Santos (2012) are not explained by inclusion criteria, but a different sample of studies were selected. A high risk of bias was identified in Santos (2012). Prioritise more recent meta-analysis.
All carbohydrate	LDL cholesterol	Diabetes	Direction of effect and statistical significance	No difference in effects of carbohydrates on LDL cholesterol in diabetes patients compared with the general population are observed.	Differences between studies may be due to inclusion criteria between studies. Differences between studies and wide confidence intervals indicates a weakness in the data for the association between carbohydrates and cholesterol in diabetes.
All carbohydrate	HbA1c	Diabetes	Statistical significance	Carbohydrates have negative effect on HbA1c in all populations but the relationship is not statistically significant.	Differences between studies may be due to inclusion criteria between studies. Differences between studies and wide confidence intervals indicates a weakness in the data for the association between carbohydrates and HbA1c in diabetes.
Fibre	Systolic blood pressure	General population	Statistical significance	Fibre decreases systolic blood pressure and the effect is statistically significant.	Hartley (2016) and Reynolds (2019) are high quality studies and the difference in statistical significance may be due to more recent evidence in Reynolds et al. Prioritise more recent evidence.
Fibre	Total cholesterol	General population	Statistical significance	Fibre decreases total cholesterol and the effect is statistically significant	Hartley (2016) and Reynolds (2019) are high quality studies and the difference in statistical significance may be due to more recent evidence in Reynolds et al. Prioritise more recent evidence.
Fibre	HDL cholesterol	General population	Direction of effect and statistical significance	No effect of fibre on HDL cholesterol	Conflicting direction of effect from two high quality studies suggests high uncertainty in evidence of fibre on HDL cholesterol.

Sugars	HBA1c	General population	Direction of effect and statistical significance	No effect of sugars on HbA1c	Two analyses repot statistically significant result for fructose. However, recent Schwingshckl (2020) review does not support this.
